# Effects of Liraglutide and Semaglutide on Cardiometabolic Dysregulation and Oxidative Stress in an Experimental Model of Metabolic Syndrome

**DOI:** 10.3390/medsci14050590

**Published:** 2026-09-19

**Authors:** Marko P. Ravić, Ivan M. Srejović, Marijana M. Andjić, Maja D. Murić, Jasmina Z. Sretenović, Nevena S. Jeremić, Isidora M. Milosavljević, Miona Lj. Vuletić, Nemanja N. Murić, Katarina R. Ravić, Sergey B. Bolevich, Svetlana P. Sergeeva, Alexander A. Gorbunov, Stefani S. Bolevich, Vladimir Lj. Jakovljević, Jovana N. Novaković

**Affiliations:** 1 Department of Pharmacy, Faculty of Medical Sciences, University of Kragujevac, Svetozara Markovica 69, 34000 Kragujevac, Serbiamarijana.andjic@fmn.kg.ac.rs (M.M.A.); njeremic@fmn.kg.ac.rs (N.S.J.); isidora.milosavljevic@fmn.kg.ac.rs (I.M.M.); jovana.novakovic@fmn.kg.ac.rs (J.N.N.); 2 Center of Excellence for the Study of Redox Balance in Cardiovascular and Metabolic Disorders, University of Kragujevac, Svetozara Markovica 69, 34000 Kragujevac, Serbia; majanikolickg90@gmail.com (M.D.M.); drvladakgbg@yahoo.com (V.L.J.); 3 Department of Physiology, Faculty of Medical Sciences, University of Kragujevac, Svetozara Markovica 69, 34000 Kragujevac, Serbia; 4 Department of Pharmacology, First Moscow State Medical University I.M. Sechenov, Trubetskaya Street 8, Str. 2, Moscow 119991, Russia; gorbunov_a_a@staff.sechenov.ru (A.A.G.); alistra555@mail.ru (S.S.B.); 5 A.P. Neyubin Institute of Pharmacy, First Moscow State Medical University I.M. Sechenov, Moscow 119435, Russia; 6 Department of Dentistry, Faculty of Medical Sciences, University of Kragujevac, Svetozara Markovica 69, 34000 Kragujevac, Serbia; miona91kg@gmail.com; 7 Department of Psychiatry, Faculty of Medical Sciences, University of Kragujevac, 34000 Kragujevac, Serbia; nmuric91@gmail.com; 8 Psychiatric Clinic, University Clinical Center Kragujevac, 34000 Kragujevac, Serbia; 9 Department for Nuclear Medicine, University Clinical Center Kragujevac, 34000 Kragujevac, Serbia; ravickaca@gmail.com; 10 Department of Pathophysiology, First Moscow State Medical University I.M. Sechenov, Moscow 119435, Russia; bolevich2011@yandex.ru (S.B.B.); sergeeva_s_p@staff.sechenov.ru (S.P.S.); 11 Faculty of Medicine, University of Banja Luka, 78000 Banja Luka, The Republic of Srpska, Bosnia and Herzegovina

**Keywords:** metabolic syndrome, myocardial ischemia/reperfusion, cardioprotection, liraglutide, semaglutide, cardiac remodeling, oxidative stress

## Abstract

Background/Objectives: Metabolic syndrome (MetS) increases susceptibility to myocardial ischemia/reperfusion (I/R) injury through metabolic disturbance, hypertension, and oxidative stress. This study aimed to compare the effects of liraglutide and semaglutide on post-ischemic cardiac function, oxidative stress, and histomorphological changes in rats with MetS. Methods: MetS was induced in male Wistar rats by a high-fat diet followed by low-dose streptozotocin. After confirmation of MetS, animals were treated with saline, liraglutide, or semaglutide for 6 weeks. Blood pressure, glycemia, oral glucose tolerance, and lipid profile were assessed during the protocol. In vivo cardiac function was evaluated by echocardiography, whereas ex vivo I/R injury was induced using the Langendorff technique. Cardiodynamic parameters, coronary flow, oxidative stress markers, and histological changes in the heart, liver, and pancreas were analyzed. Results: Both liraglutide and semaglutide improved the cardiometabolic profile of MetS rats, with semaglutide showing a more evident effect on body weight control. In the Langendorff model, both treatments improved post-ischemic recovery of myocardial contractility and relaxation during reperfusion. Treated animals also showed a more favorable oxidative stress profile, particularly lower superoxide anion levels and enhanced antioxidant defense. Histologically, both agents attenuated myocardial hypertrophy and collagen deposition, improved hepatic architecture by reducing inflammatory changes, and preserved pancreatic structure with less lipid accumulation and tissue injury. Conclusions: Liraglutide and semaglutide exerted significant cardioprotective and tissue-protective effects in experimental MetS complicated by myocardial I/R injury. These findings support their potential in limiting post-ischemic cardiac dysfunction and multiorgan damage in metabolically compromised conditions.

## 1. Introduction

Metabolic syndrome (MetS) is a pathophysiological state characterized by the convergence of obesity, impaired glucose regulation, dyslipidemia, hypertension, insulin resistance, endothelial dysfunction, and chronic low-grade inflammation, creating a pro-atherogenic, pro-inflammatory, and pro-oxidative environment that increases cardiovascular risk [1,2,3,4]. Oxidative stress and endothelial injury are considered important links between metabolic dysregulation and cardiovascular damage [4]. In such a background, the myocardium becomes particularly vulnerable to ischemic stress. The pathological basis of myocardial ischemia is most commonly an acute reduction or interruption of coronary blood flow caused by thrombosis superimposed on an atherosclerotic plaque [5]. Oxygen deprivation rapidly disrupts cardiomyocyte energy balance, leading to adenosine triphosphate (ATP) depletion, intracellular acidosis, impaired ionic homeostasis, and calcium overload [6]. If prolonged, these disturbances culminate in necrotic and apoptotic cell death and determine the extent of post-ischemic structural and functional impairment [7]. Although reperfusion is essential for tissue salvage, it can paradoxically induce additional damage, known as ischemia/reperfusion (I/R) injury [8]. I/R injury has become one of the focal points in experimental cardiology. Excessive reactive oxygen species (ROS) generation, mitochondrial dysfunction, calcium overload, inflammation, and microcirculatory impairment contribute to I/R injury [9]. In MetS, pre-existing oxidative stress, endothelial dysfunction, dyslipidemia, and inflammation may further aggravate these mechanisms, potentially increasing myocardial vulnerability to I/R injury [2,10,11].

Within this framework, glucagon-like peptide-1 (GLP-1) receptor agonists (GLP-1RAs) have attracted increasing attention. Initially developed for the treatment of type 2 diabetes, these agents have demonstrated broader metabolic and cardiovascular effects that extend beyond glycemic control [12]. GLP-1 signaling influences glucose homeostasis, appetite regulation, and energy balance, while experimental and clinical studies increasingly suggest favorable effects on vascular function, oxidative stress, and tissue protection in cardiometabolic disease [13]. Semaglutide also showed hepatoprotective effects and the benefit of its use in people with metabolic dysfunction-associated steatohepatitis (MASH) and liver fibrosis has been confirmed [14]. Among GLP-1RAs, liraglutide and semaglutide are of particular interest because of their established metabolic and cardiovascular benefits, although their potential cardioprotective mechanisms in MetS-associated myocardial I/R injury remain incompletely understood [15].

Besides the known effects of both investigated drugs on improvement of glucose utilization and metabolic functions, their cardioprotective potential remains unclear, as well as effects on cardiac and systemic redox balance during I/R injury. Therefore, in this study, we focused on cardiometabolic changes and the potential of liraglutide and semaglutide in reducing the consequences caused by ischemia and reperfusion in the field of MetS. Furthermore, we wanted to compare the effects on heart protection of liraglutide and semaglutide in in vivo and ex vivo experimental protocols in rats with MetS subjected to myocardial I/R. Given that MetS affects not only metabolic balance but also redox status, cardiovascular performance, and structural integrity of multiple organs, we evaluated oxidative stress parameters, echocardiographic findings, ex vivo cardiodynamic responses, and histological alterations in the heart, liver, and pancreas. Such an approach was intended to provide a broader view of the cardiometabolic and multiorgan effects of these two GLP-1RAs in a model that closely reflects the interplay between metabolic syndrome and I/R injury.

## 2. Materials and Methods

### 2.1. Experimental Animals and Study Design

This chronic experimental study was performed in vivo and ex vivo at the Center of Excellence for the Study of Redox Balance in Cardiovascular and Metabolic Disorders, Faculty of Medical Sciences, University of Kragujevac, Serbia. All experimental procedures were conducted in accordance with the EU Directive for the Protection of Vertebrate Animals used for Experimental and Other Scientific Purposes 86/609/EEC and approved by the Ethics Committee for the Welfare of Experimental Animals of the Faculty of Medical Sciences, University of Kragujevac (approval No. 01-11876/4, date: 11 October 2019).

Male Wistar Albino rats, 4 weeks old and weighing 100 ± 20 g, were obtained from the Military Medical Academy, Belgrade, Serbia. Animals were housed under controlled laboratory conditions (room temperature 21 ± 2 °C, humidity 55 ± 5%, and 12-h dark–light cycle) with free access to food and water.

**Figure 1:**
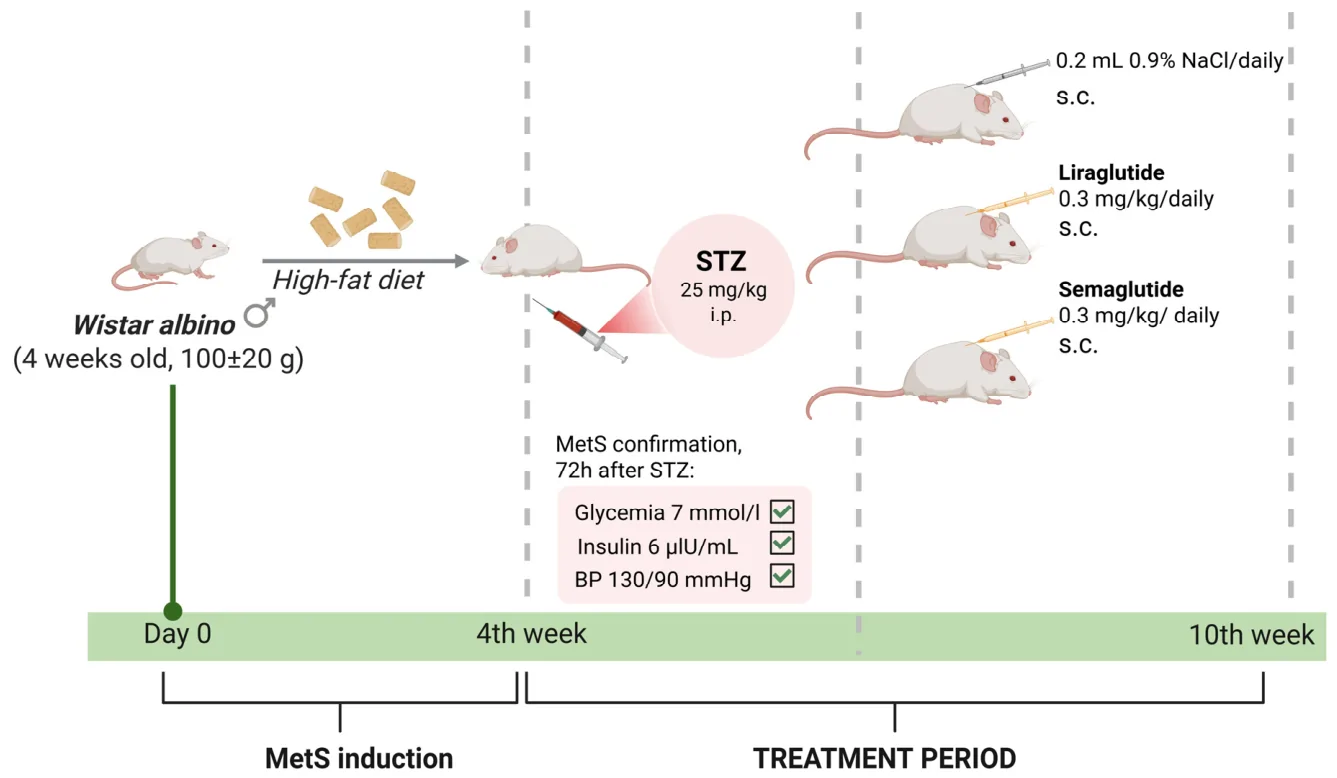
Schematic representation of experimental protocol design. Created in BioRender. Srejovic, I. (2026) https://BioRender.com/4sk383o (accessed on 14 September 2026).

A total of 35 rats were initially included in the study. Five animals were excluded after streptozotocin administration because fasting glycemia, insulin, or blood pressure values did not meet the predefined criteria for metabolic syndrome [16]. The remaining 30 animals were randomly allocated to the groups analyzed in the present study (10 animals with confirmed MetS per group) (Figure 1): (1) MetS control group, treated subcutaneously with 0.9% NaCl; (2) liraglutide-treated group, receiving 0.3 mg/kg/day subcutaneously; and (3) semaglutide-treated group, receiving 0.3 mg/kg/day subcutaneously. Doses for liraglutide and semaglutide were chosen according to the previous studies on similar design [17,18].

### 2.2. Induction of Metabolic Syndrome

MetS was induced using a high-fat diet/low-dose streptozotocin protocol [16]. Briefly, rats were fed a high-fat diet for 4 weeks containing 30% fat, 10% protein, 55% starch, and 5% fiber. After 12 h of fasting, streptozotocin was administered intraperitoneally at a dose of 25 mg/kg. Seventy-two hours later, fasting blood glucose, fasting insulin, and blood pressure were measured. Metabolic syndrome was considered established in animals with fasting glycemia > 7 mmol/L, fasting insulin > 6 µIU/L, and blood pressure > 130/90 mmHg (Figure 1) [15]. These metabolic variables are crucial for confirmation of MetS and are commonly used in studies of similar design, conducted by us and others, and described in the review literature [19,20,21].

### 2.3. Chronic Treatment and Metabolic Assessment

Following confirmation of MetS, animals received saline, liraglutide, or semaglutide for 6 weeks. Body weight and fasting blood glucose (after 12 h of fasting; Accu-Chek glucometer, Roche Diagnostics, Indianapolis, IN, USA) were recorded before treatment initiation and weekly thereafter. Three predefined time points were used for the statistical analysis and presentation of these variables and of systolic blood pressure: baseline, before the first dose (designated 0), the end of week 3 of treatment (3), and the end of week 6 of treatment, corresponding to the end of the protocol (6).

At the end of the protocol, an oral glucose tolerance test (OGTT) was performed after overnight fasting. Blood samples were collected from the tail vein. Immediately after baseline glucose determination, glucose was administered by oral gavage at a dose of 2 g/kg of body weight. Glycemia was measured at 0, 30, 60, 90, 120, and 180 min, whereas insulin levels were determined at 0 and 180 min using a commercial enzyme-linked immunosorbent assay (ELISA). After sacrifice, blood was collected for determination of total cholesterol and triglycerides using an automated biochemical analyzer (Dimension Xpand, Siemens, IL, USA) and commercial reagent kits [16].

### 2.4. Hemodynamic Measurements and Echocardiography

Systolic blood pressure was measured at the same three time points (0, 3, and 6), before treatment initiation (0), after three weeks (3), and at the end of the protocol (6) using the tail-cuff method (Rat Tail Cuff Method Blood Pressure Systems, MRBP-R, IITC Life Science Inc., Los Angeles, CA, USA). To minimize stress-related variation, animals were acclimatized to the procedure before each measurement session, and the mean of six consecutive recordings was used for analysis.

Transthoracic echocardiography was performed in the final week of the study using a Hewlett-Packard Sonos 5500 ultrasound system (Andover, MA, USA) equipped with a 15.0 MHz transducer. All animals were anesthetized with isoflurane inhalation anesthesia prior to imaging. Anesthesia was induced using 5% isoflurane in the anesthesia induction box. After achieving anesthesia, the isoflurane concentration, during the echocardiography procedure, was reduced to 1.5% and applied via a mask for rats. Heart rate during echocardiography procedures varied between 280 and 350 bpm. The following M-mode parameters were recorded: interventricular septal thickness at end-diastole and end-systole (IVSd and IVSs, respectively), left ventricular internal diameter at end-diastole and end-systole (LVIDd and LVIDs, respectively), and left ventricular posterior wall thickness at end-diastole and end-systole (LVPWd and LVPWs, respectively). Fractional shortening (FS) was calculated as follows: FS (%) = 100 × (LVIDd − LVIDs)/LVIDd. Ejection fraction (EF) was calculated using the Teichholz formula [22].

### 2.5. Ischemia/Reperfusion Protocol

At the end of the treatment protocol, rats were sacrificed, and hearts were rapidly excised and mounted on a Langendorff apparatus (Experimetria Ltd., Budapest, Hungary) for retrograde perfusion. Hearts were perfused with Krebs–Henseleit solution continuously gassed with 95% O_2_ and 5% CO_2_ and maintained at 37 °C, pH 7.4.

A pressure transducer introduced into the left ventricle enabled continuous monitoring of the following cardiodynamic parameters: maximum and minimum rates of left ventricular pressure development (dp/dt max and dp/dt min, respectively), systolic and diastolic left ventricular pressure (SLVP and DLVP, respectively), and heart rate (HR). Coronary flow (CF) was measured flowmetrically.

After a stabilization period, global ischemia was induced for 30 min, followed by 60 min of reperfusion. Cardiodynamic parameters and coronary flow were recorded at the end of stabilization and at 1, 3, 5, 10, 15, 30, 45, and 60 min of reperfusion [23]. Three predefined time points were used for statistical analysis and presentation: the end of the stabilization period (S), the 1st minute of reperfusion (1′), and the 60th minute of reperfusion (60′). Coronary venous effluent (CVE) was collected at the same three time points for the determination of oxidative stress markers. This notation (S, 1′, 60′) is used throughout the Results and in all figures.

### 2.6. Oxidative Stress Assessment

Oxidative stress was evaluated spectrophotometrically in plasma, CVE, and erythrocyte lysates using a Shimadzu UV-1800 spectrophotometer (Kyoto, Japan). Pro-oxidative markers included thiobarbituric acid reactive substances (TBARS), nitrites (NO_2_^−^), superoxide anion radical (O_2_^−^), and hydrogen peroxide (H_2_O_2_), whereas antioxidant status was assessed by measuring superoxide dismutase (SOD), catalase (CAT), and reduced glutathione (GSH).

#### 2.6.1. Determination of Index of Lipid Peroxidation (TBARS)

Lipid peroxidation was estimated as TBARS in plasma and CVE. For CVE, 800 μL of sample was mixed with 200 μL of 1% thiobarbituric acid (TBA) in 0.05 NaOH, incubated at 100 °C for 15 min, and read at 530 nm. Plasma samples were precipitated with 28% trichloroacetic acid, centrifuged, and the supernatant was then reacted with TBA under the same conditions. Krebs–Henseleit solution and distilled water were used as blanks for coronary effluent and plasma, respectively [23].

#### 2.6.2. Determination of Nitrites

Nitrite concentration, used as an indirect marker of nitric oxide production, was determined in plasma and CVE using the Griess reaction. CVE was mixed with freshly prepared Griess reagent and NO buffer, whereas plasma samples were first deproteinized with perchloric acid and ethylenediaminetetraacetic acid (EDTA), centrifuged, neutralized with potassium carbonate, and then processed in the same manner. Absorbance was measured at 550 nm, and sodium nitrite was used for calibration [16].

#### 2.6.3. Determination of Superoxide Anion Radical

Superoxide anion radical (O_2_^−^) levels were measured in plasma and CVE by reduction in nitro blue tetrazolium (NBT). Briefly, 50 μL of sample was added to 950 μL of assay mixture containing Tris-HCl buffer, EDTA, gelatin, and NBT, and absorbance was recorded after repeated mixing at 60 s intervals [23].

#### 2.6.4. Determination of Hydrogen Peroxide

H_2_O_2_ concentration was determined by phenol red oxidation in the presence of horseradish peroxidase. Briefly, 800 μL of phenol red solution was mixed with 200 μL of sample and 10 μL of peroxidase, incubated for 10 min at room temperature, and measured spectrophotometrically [23].

#### 2.6.5. Determination of Superoxide Dismutase Activity

SOD activity was determined in erythrocyte lysates by the epinephrine method according to Misra. This assay is based on the ability of SOD to inhibit epinephrine auto-oxidation in alkaline medium by scavenging superoxide anion radicals. Briefly, 100 μL of erythrocyte lysate was mixed with 1 mL of carbonate buffer, after which 100 μL of epinephrine was added. Absorbance was measured spectrophotometrically at 470 nm [16].

#### 2.6.6. Determination of Reduced Glutathione

GSH concentration was measured in erythrocyte lysates according to the Beutler method using 5,5′-dithiobis-2-nitrobenzoic acid (DTNB). In brief, 50 μL of hemolysate was mixed with 200 μL of 0.1% EDTA and 385 μL of precipitation buffer. After incubation on ice for 15 min and centrifugation at 4000 rpm for 10 min, 300 μL of the obtained supernatant was mixed with 750 μL of sodium phosphate and 100 μL of DTNB. Following 10 min of incubation, absorbance was recorded at 412 nm [16].

#### 2.6.7. Determination of Catalase Activity

CAT activity was determined in erythrocyte lysates by monitoring the decomposition of hydrogen peroxide. Before analysis, the lysate was diluted with distilled water and ethanol. Thereafter, 100 μL of diluted sample was mixed with 50 μL of catalase buffer and 1 mL of 10 mM hydrogen peroxide. Absorbance was measured spectrophotometrically at 360 nm, and enzyme activity was expressed according to the applied assay protocol [16].

### 2.7. Histological and Morphometric Analysis

After completion of the experimental protocol, including ex vivo assessment on the Langendorff apparatus, the organs of interest were isolated and fixed in 4% neutral paraformaldehyde for 24 h. The samples were then dehydrated through graded ethanol concentrations, cleared in xylene, and embedded in Histowax^®^ paraffin medium (Histolab Product AB, Göteborg, Sweden). Paraffin blocks were cut into 5 µm thick sections using a rotary microtome (RM 2125RT, Leica Microsystems, Wetzlar, Germany).

Tissue sections were stained with hematoxylin and eosin (HE) for the evaluation of general morphology and structural alterations. Picrosirius Red staining was used for the assessment of collagen deposition. With this method, collagen fibers are stained red, and muscle fibers and cytoplasm appear yellowish, while nuclei are stained dark brown to black.

Histological images were obtained using a digital camera connected to an Olympus BX51 light microscope. Morphometric analysis was performed using calibrated Axiovision software 4.6 (Zeiss, White Plains, NY, USA) and Image-Pro Plus software 7.0 (Media Cybernetics, Rockville, MD, USA), according to previously described methodology [16]. Cardiomyocyte cross-sectional area was measured in at least 100 cardiomyocytes per animal. Results are presented in μm^2^. Collagen content was expressed as the percentage of collagen-positive area relative to the total analyzed tissue area. The diameter of hepatocytes was determined by measuring the widest part of each hepatocyte. Hepatocytes with clearly visible cell boundaries were selected for analysis, whereas cells with indistinct boundaries were excluded from the measurements. The diameter of the islets of Langerhans was also determined [24,25]. Results are presented in μm.

### 2.8. Statistical Analysis

Statistical analysis was performed using GraphPad Prism 9.5.0 for Windows (GraphPad Software, Boston, MA, USA). Data normality was assessed using the Shapiro–Wilk test, and results are presented as mean ± standard deviation (SD). Parameters measured once per animal at the end of the protocol (serum lipids, echocardiographic indices, and systemic oxidative stress markers) were compared among the three groups by one-way ANOVA followed by Tukey’s multiple-comparisons test.

Parameters measured repeatedly in the same animals over time—body weight, fasting glycemia, and systolic blood pressure at 0, 3, and 6 weeks of treatment; glucose and insulin during the OGTT; and cardiodynamic parameters and CVE oxidative stress markers at S, 1′, and 60′—were analyzed by two-way ANOVA with repeated measures on the time factor (treatment × time). When individual data points were missing, a mixed-effects model (restricted maximum likelihood) as implemented in GraphPad Prism was applied instead. Between-group differences at each time point and within-group differences across time points were subsequently assessed using the Šídák multiple-comparisons test. Exact adjusted *p*-values are reported for all statistically significant comparisons. The statistical test, the number of animals analyzed, and the meaning of the symbols are stated in each figure legend and in the footnote to Table 1. A *p* value < 0.05 was considered statistically significant.

The sample size and number of animals per group were determined using G*Power 3.1.9.7, with α = 0.05 and statistical power set at 0.8, based on data from studies of similar design [19].

## 3. Results

### 3.1. Effects of GLP-1RAs on Body Weight, Glycemia, and Insulin Levels

**Figure 2:**
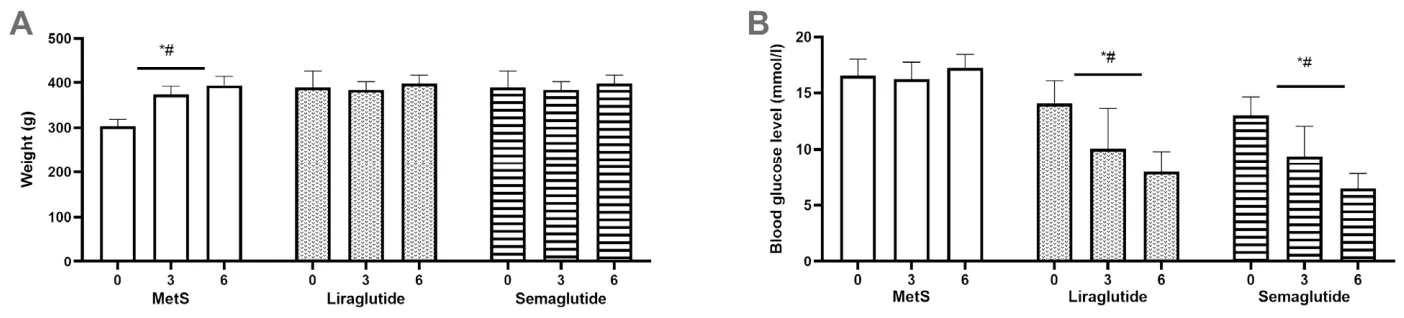
Effects of liraglutide and semaglutide on body weight and fasting blood glucose during treatment. (**A**) Body weight; (**B**) fasting blood glucose; 0—baseline, before treatment initiation; 3—week 3 of treatment; 6—week 6 of treatment. Within-group comparisons between time points: * *p* < 0.05, 0 vs. 3; # *p* < 0.05, 0 vs. 6. Two-way repeated-measures ANOVA with Šídák multiple-comparisons test. Values are presented as mean ± SD, n = 10 per group.

During the experimental period, a statistically significant increase in body weight was observed in the MetS group after the third (*p* < 0.0001) and sixth week (*p* < 0.0001) of the protocol. Treatment with liraglutide and semaglutide resulted in no changes at both time points compared with baseline values (Figure 2A).

Figure 2B shows blood glucose levels with statistically significant intra-group changes over time. In the MetS group, blood glucose levels did not change significantly during the experimental protocol. In contrast, animals treated with liraglutide and semaglutide exhibited a statistically significant glucose-lowering effect after six weeks compared with baseline values (*p* = 0.0001 and *p* < 0.0001, respectively). The decreasing trend in glycemic values correlated with the duration of treatment in both the liraglutide- and semaglutide-treated groups (Figure 2B).

**Figure 3:**
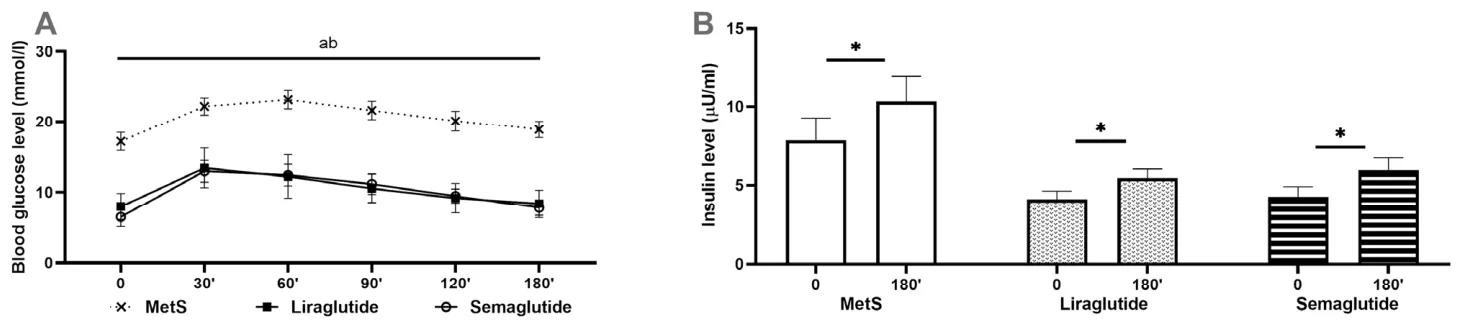
Effects of liraglutide and semaglutide on blood glucose and insulin during the OGTT. (**A**) Blood glucose at 0′ (fasting) and 30′, 60′, 90′, 120′, and 180′ after glucose administration; (**B**) insulin at 0′ and 180′. Between-group comparisons: a *p* < 0.05 vs. MetS; b *p* < 0.05 liraglutide vs. semaglutide. In (**B**), * *p* < 0.05, 0′ vs. 180′ within the same group. Two-way repeated-measures ANOVA with Šídák multiple-comparisons test. Values are presented as mean ± SD, n = 10 per group.

Figure 3A,B present glucose and insulin levels measured during the OGTT. As shown in Figure 3A, glycemic values in the MetS group were significantly higher than those observed in the liraglutide- and semaglutide-treated groups at all measured time points (0, 30, 60, 90, 120, and 180 min; all comparisons *p* < 0.0001). In both experimental groups, glucose levels returned close to baseline values by 180 min, whereas in the MetS group, glycemic levels remained elevated at 180 min compared to baseline, although this difference did not reach statistical significance. Figure 3B shows insulin levels measured at 0 and 180 min of the OGTT. A statistically significant increase in insulin levels at 180 min compared to baseline was observed in all groups (MetS, *p* = 0.0241; liraglutide, *p* = 0.0105; semaglutide, *p* = 0.0028). Furthermore, insulin levels in the MetS group were consistently higher at both time points compared to the liraglutide- and semaglutide-treated groups.

### 3.2. Effects of GLP-1RAs on Lipid Status

**Figure 4:**
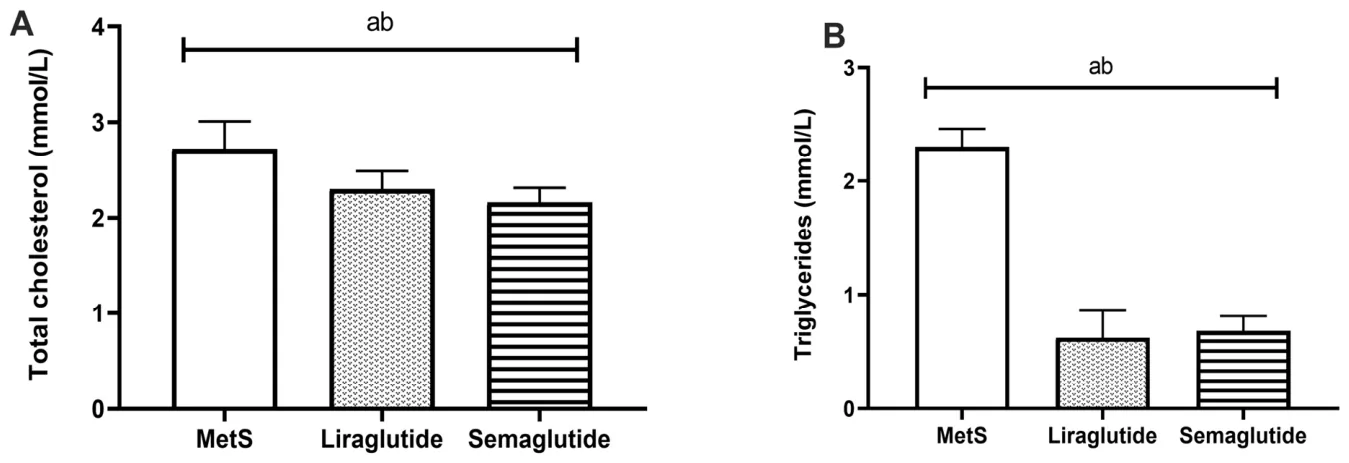
Effects of liraglutide and semaglutide on total cholesterol and triglycerides. (**A**) Total cholesterol; (**B**) triglycerides. Between-group comparisons: statistical significance at the level of *p* < 0.05. a—MetS vs. liraglutide; b—MetS vs. semaglutide;. One-way ANOVA with Tukey’s multiple-comparisons test. Values are presented as mean ± SD.

Lipid status was assessed based on total cholesterol and triglyceride levels, as presented in Figure 4. A statistically significant reduction in total cholesterol levels was observed in rats treated with liraglutide (*p* = 0.0243) and semaglutide (*p* = 0.0039) compared to the MetS group (Figure 4A). Similarly, triglyceride levels were significantly decreased in the groups treated with GLP-1R agonists compared to the untreated MetS group (both *p* < 0.0001) (Figure 4B).

### 3.3. Effects of GLP-1RAs on Hemodynamic Measurements and Echocardiography

**Figure 5:**
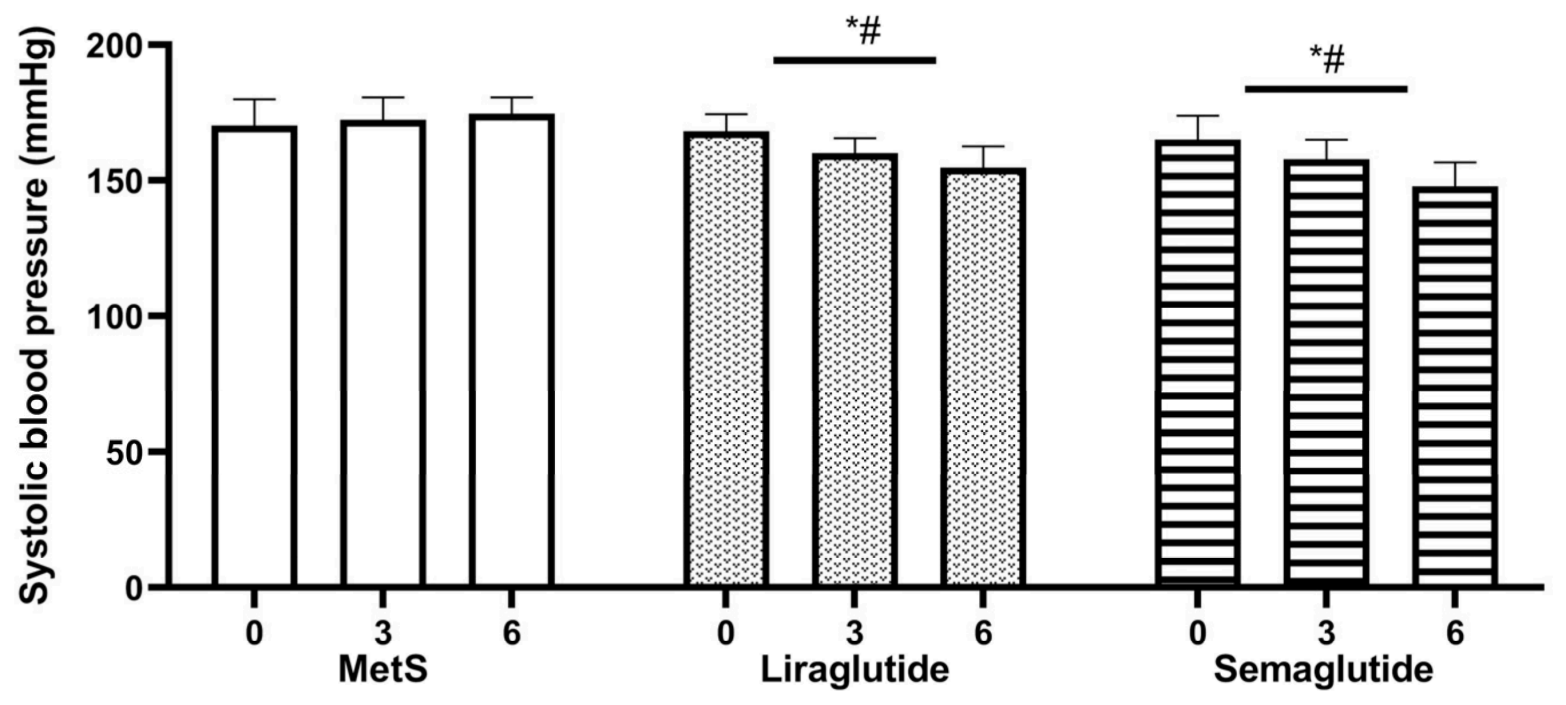
Effects of liraglutide and semaglutide on systolic blood pressure during treatment. 0—baseline, before treatment initiation; 3—week 3 of treatment; 6—week 6 of treatment. Within-group comparisons across time: statistical significance at the level *p* < 0.05 * 0 vs. 3; # 0 vs. 6. Two-way repeated-measures ANOVA with Šídák multiple-comparisons test. Values are mean ± SD, n = 10 per group.

Systolic blood pressure was measured at different time intervals to assess potential treatment-dependent changes. The obtained values are presented in Figure 5. In animals treated with GLP-1 receptor agonists, lower systolic blood pressure values were observed after three and six weeks of treatment compared with baseline values (liraglutide, *p* = 0.0271 and *p* = 0.0098; semaglutide, *p* = 0.0398 and *p* = 0.0098, respectively), and in the semaglutide-treated group, systolic blood pressure was further reduced at the sixth week compared with the third week (*p* = 0.0167). On the other hand, systolic blood pressure in the MetS group remained relatively stable throughout the experimental protocol (Figure 5).

Echocardiographic parameters, including left ventricular dimensions, EF, and FS, are presented in Table 1. In rats treated with liraglutide and semaglutide, a significant reduction in IVSd (both *p* < 0.0001) was observed compared to the MetS group. On the other hand, LVIDd (*p* = 0.0003) and LVIDs were significantly increased in the semaglutide treatment group relative to the MetS group and liraglutide group. Additionally, a significant difference in LVIDs (*p* < 0.031) was detected between the liraglutide-treated group and the MetS group.

### 3.4. Effects of GLP-1RAs on Cardiodynamic Parameters

**Figure 6:**
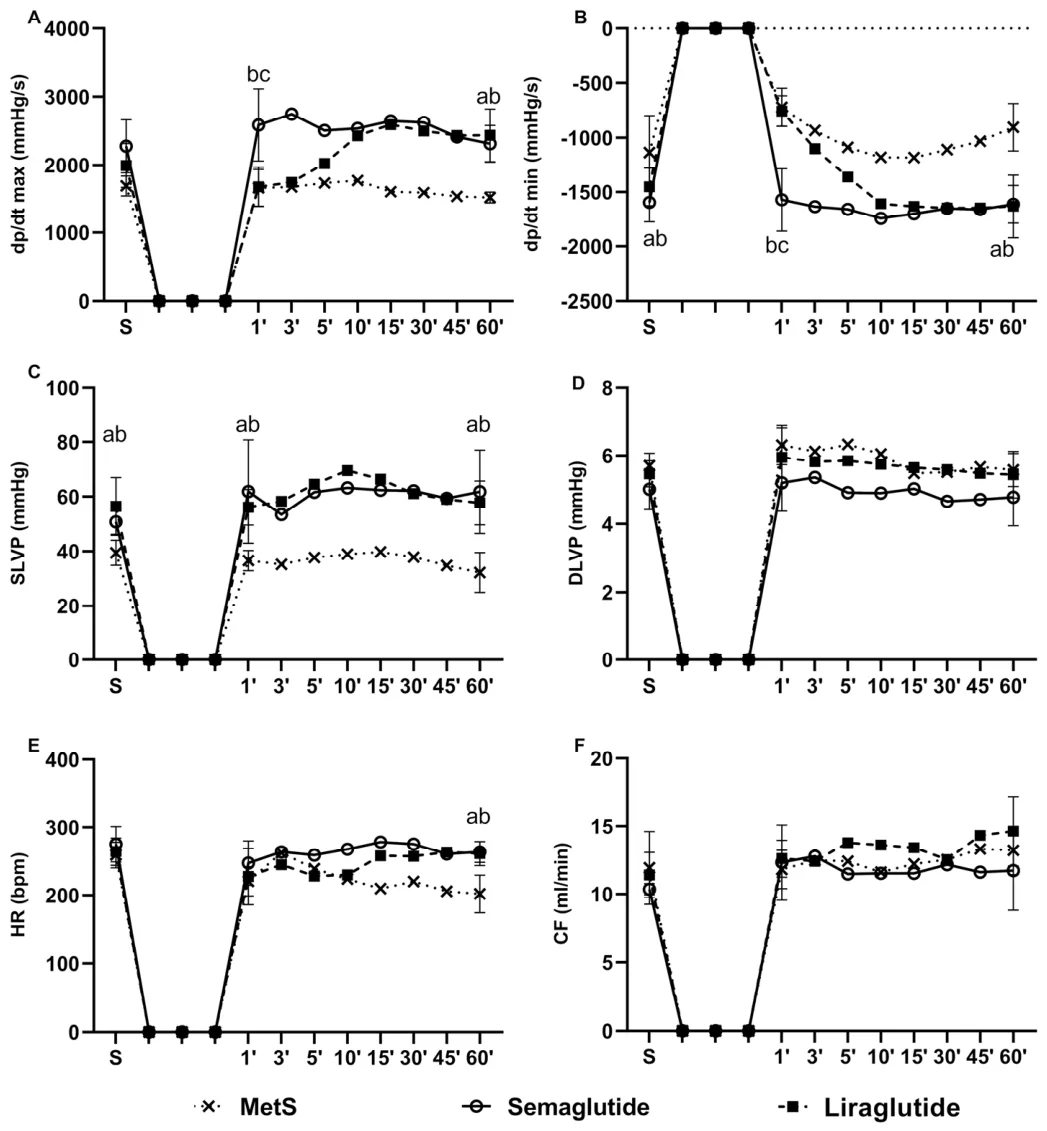
Effects of liraglutide and semaglutide on cardiodynamic parameters between experimental groups. (**A**) dp/dt max—the maximum rate of LV pressure development, (**B**) dp/dt min—minimum rate of LV pressure development, (**C**) SLVP—systolic LV pressure, (**D**) DLVP—diastolic LV pressure, (**E**) HR—heart rate, and (**F**) CF—coronary flow. Between-group comparisons at three time points. S—end of the stabilization period; 1′ and 60′—1st and 60th minute of reperfusion. a—MetS vs. liraglutide; b—MetS vs. semaglutide; c—liraglutide vs. semaglutide. Statistical significance at the level of *p* < 0.05. Two-way repeated-measures ANOVA with Šídák multiple-comparisons test. Values are presented as mean ± SD, n = 10 per group.

Cardiodynamic parameters are presented in Figure 6. Six-week treatment with liraglutide or semaglutide generally improved cardiac function compared with untreated MetS animals. For dp/dt max, semaglutide produced an early reperfusion improvement compared with both MetS (*p* = 0.0006) and liraglutide (*p* = 0.0008), while at 60 min of reperfusion both liraglutide (*p* = 0.0003) and semaglutide (*p* = 0.0046) showed higher values than MetS, with no difference between treatments. No differences were observed during stabilization (S) (Figure 6A).

Regarding diastolic function (dp/dt min), both treated groups exhibited improved recovery relative to MetS animals, with dp/dt min values during late reperfusion (liraglutide, *p* < 0.0001; semaglutide, *p* = 0.0002), indicating improved myocardial relaxation. This improvement was already evident during the stabilization (S) (liraglutide, *p* < 0.0041; semaglutide, *p* = 0.0032) period and remained pronounced during late reperfusion. At the 1st minute of reperfusion, dp/dt min was significantly more negative in the semaglutide group than in the MetS (*p* < 0.0001) and the liraglutide group (*p* < 0.0001) (Figure 6B).

For SLVP, both liraglutide and semaglutide groups showed significantly higher values at the end of stabilization (liraglutide, *p* = 0.0410; semaglutide, *p* = 0.0133) and both early (liraglutide, *p* = 0.0310; semaglutide, *p* = 0.0033) and late reperfusion (liraglutide, *p* = 0.0231; semaglutide, *p* = 0.0123) compared with the MetS group, while no significant differences were observed between the two treatment groups (Figure 6C).

No significant differences in DLVP were detected among the MetS, liraglutide, and semaglutide groups (Figure 6D).

Heart rate did not differ significantly among the groups during the stabilization period or at the 1st minute of reperfusion. At the 60th minute of reperfusion, however, heart rate was significantly higher in both the liraglutide (*p* = 0.0074) and the semaglutide-treated group (*p* = 0.0083) than in the untreated MetS group, with no significant difference between the two treatments (Figure 6E).

No significant differences in coronary flow were observed among the groups during either stabilization or reperfusion (Figure 6F).

**Figure 7:**
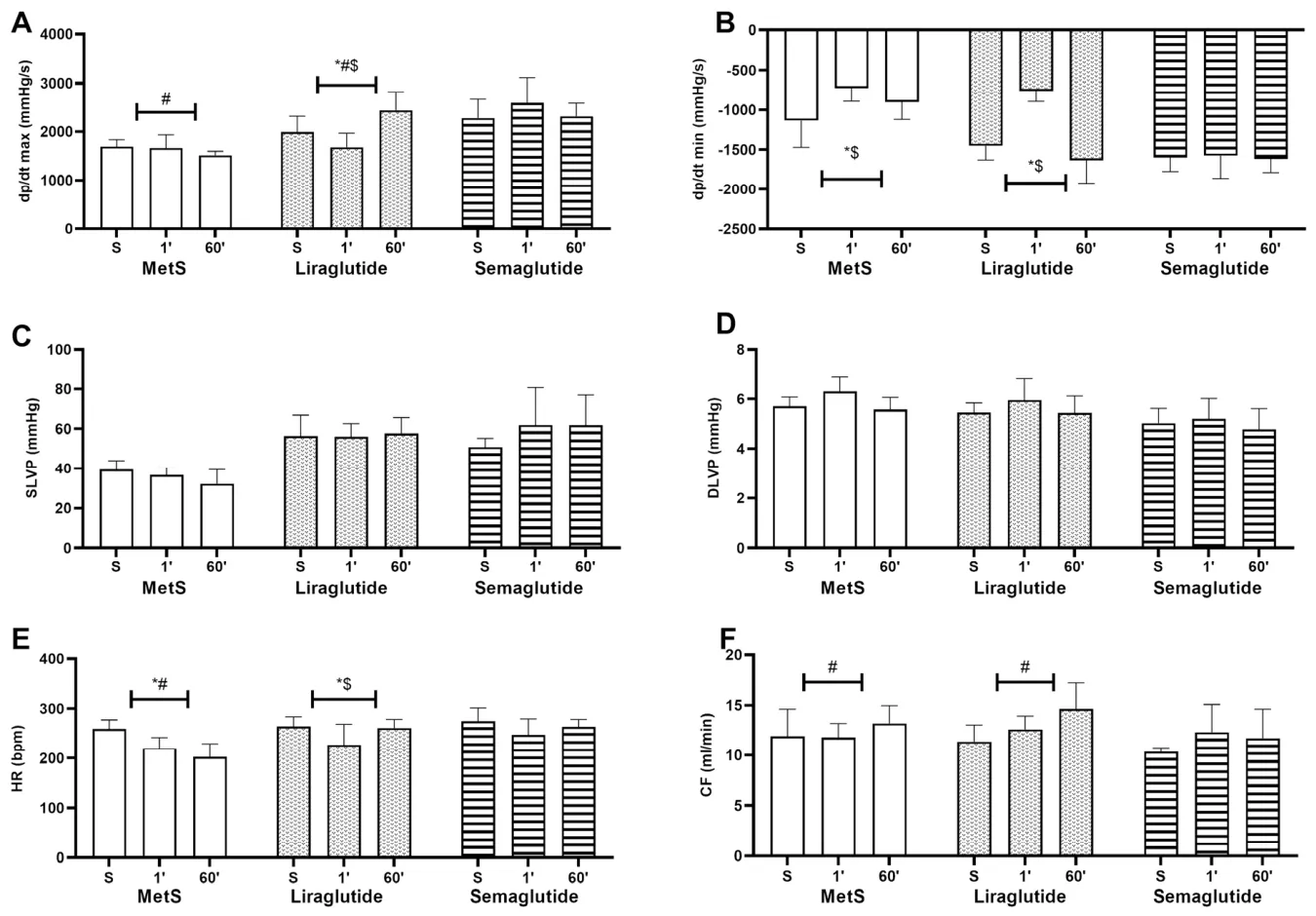
Effects of liraglutide and semaglutide on cardiodynamic parameters between specific time points in the experimental group. (**A**) dp/dt max—the maximum rate of LV pressure development, (**B**) dp/dt min—minimum rate of LV pressure development, (**C**) SLVP—systolic LV pressure, (**D**) DLVP—diastolic LV pressure, (**E**) HR—heart rate, and (**F**) CF—coronary flow. S—end of the stabilization period; 1′ and 60′—1st and 60th minute of reperfusion. Within-group comparisons between time points: * S vs. 1′; # S vs. 60′; $ 1′ vs. 60′. Statistical significance at the level of *p* < 0.05. Two-way repeated-measures ANOVA with Šídák multiple-comparisons test. Values are presented as mean ± SD, n = 10 per group.

The changes in cardiodynamic parameters across all experimental groups, assessed after the stabilization period and during early (1st minute) and late (60th minute) reperfusion, are presented in Figure 7. In untreated MetS animals, dp/dt max significantly decreased at the 60th minute of reperfusion compared with stabilization (*p* = 0.0047), whereas liraglutide treatment increased dp/dt max at the 60th minute of reperfusion compared with both stabilization (*p* = 0.0143) and early reperfusion (*p* = 0.0222), indicating improved contractile recovery (Figure 7A).

For dp/dt min, a significant transient impairment was observed in the MetS and liraglutide groups at the 1st minute of reperfusion compared with the stabilization period (MetS, *p* = 0.0023, liraglutide, *p* = 0.0002), followed by a significant recovery at the 60th minute (MetS, *p* = 0.0234, liraglutide, *p* < 0.0001) (Figure 7B).

No significant changes in SLVP or DLVP were detected between time points within any group following retrograde perfusion of isolated rat hearts (Figure 7C,D).

Heart rate decreased during early and late reperfusion in MetS animals (*p* = 0.0352 and *p* = 0.0112), while liraglutide-treated animals showed an initial decrease at the 1st minute (*p* = 0.0002) followed by recovery at the 60th minute (*p* = 0.0332) (Figure 7E).

Furthermore, six-week liraglutide treatment resulted in a significant increase in coronary flow at the 60th minute of reperfusion compared with the stabilization period (*p* = 0.0012), similar to what we see in the MetS group (*p* = 0.0443) (Figure 7F), suggesting improved coronary perfusion and functional recovery during the late phase of reperfusion.

### 3.5. Effects of GLP-1RAs on Systemic Oxidative Stress Parameters

**Figure 8:**
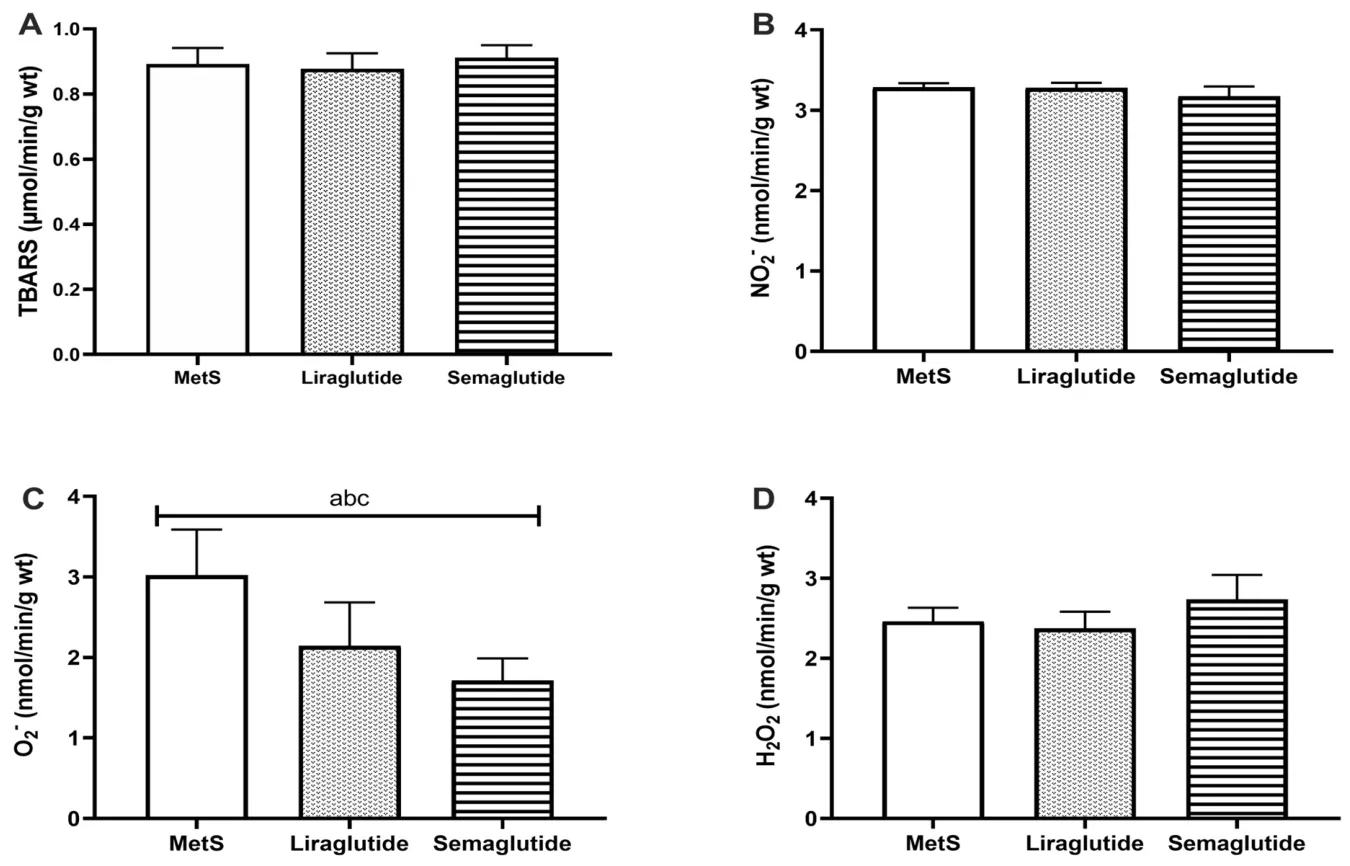
Effects of liraglutide and semaglutide on pro-oxidant parameters in plasma. (**A**) TBARS; (**B**) NO_2_^−^; (**C**) O_2_^−^; (**D**) H_2_O_2_. Between-group comparisons. a—MetS vs. liraglutide; b—MetS vs. semaglutide; c—liraglutide vs. semaglutide. Statistical significance at the level of *p* < 0.05. One-way ANOVA with Tukey’s multiple-comparisons test. Values are presented as mean ± SD, n = 10 per group.

The levels of pro-oxidative markers measured in plasma are presented in Figure 8. The results indicate that there were no statistically significant differences in the levels of TBARS, NO_2_^−^, and H_2_O_2_ between the experimental groups and the MetS group. However, six-week treatment with liraglutide and semaglutide resulted in a statistically significant reduction in O_2_^−^ levels compared to the untreated group (*p* = 0.0202 and *p* = 0.0017, respectively) (Figure 8C). Furthermore, O_2_^−^ levels were significantly lower in the semaglutide-treated group compared to the liraglutide-treated group (*p* = 0.0384).

**Figure 9:**
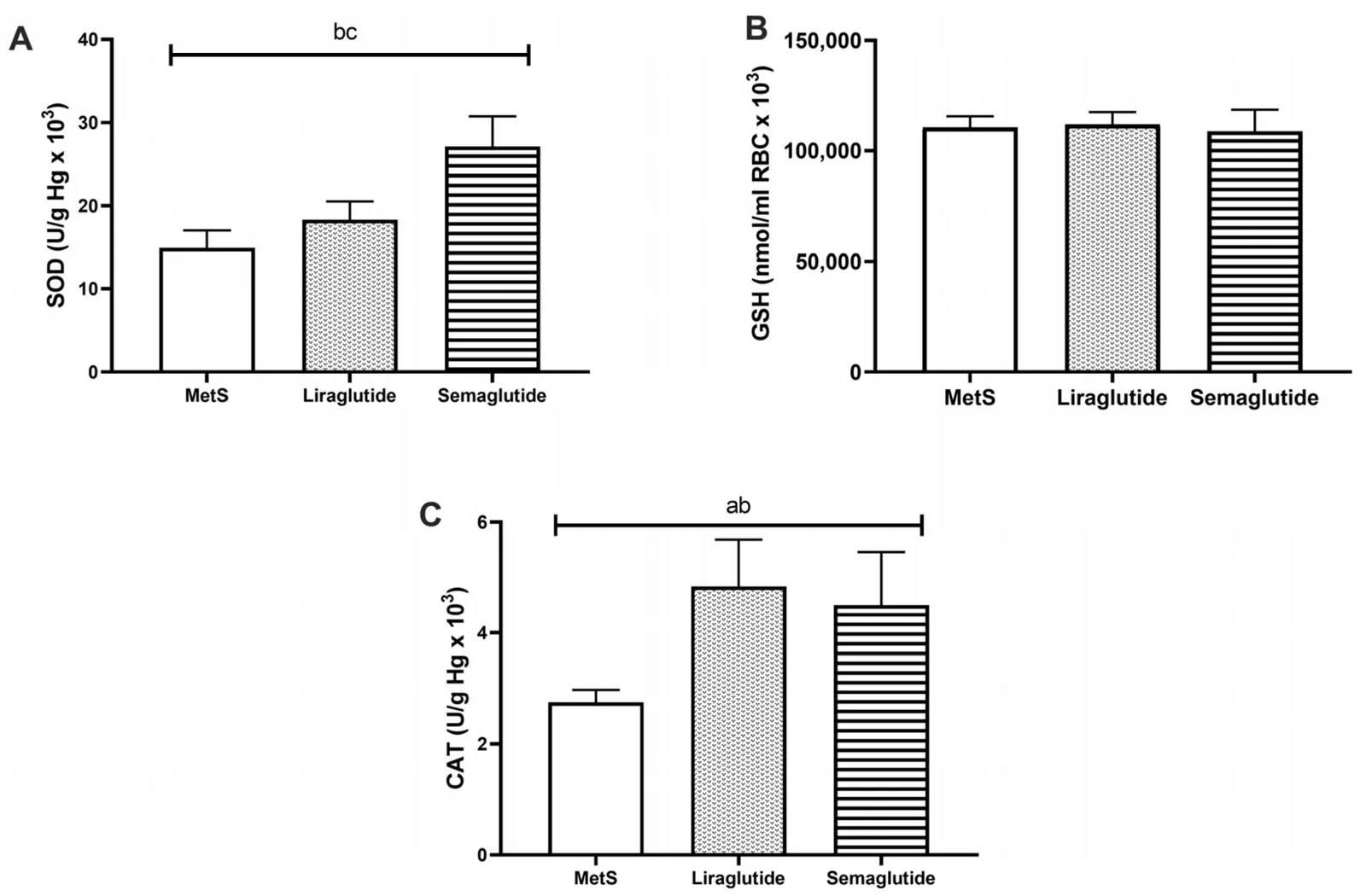
Effects of liraglutide and semaglutide on antioxidant parameters in peripheral venous blood erythrocyte lysate. (**A**) SOD; (**B**) GSH; (**C**) CAT. Between-group comparisons. a—MetS vs. liraglutide; b—MetS vs. semaglutide; c—liraglutide vs. semaglutide. Statistical significance at the level of *p* < 0.05. One-way ANOVA with Tukey’s multiple-comparisons test. Values are presented as mean ± SD, n = 10 per group.

The parameters of the antioxidant defense system are presented in Figure 9. SOD activity was significantly increased in the semaglutide-treated group compared to the MetS group (*p* < 0.0001). Additionally, SOD activity was significantly higher in the semaglutide group compared to the liraglutide group (*p* = 0.0003) (Figure 9A). The activity of CAT was significantly elevated in groups treated with GLP-1R agonists compared to the untreated MetS group (liraglutide, *p* = 0.0006; semaglutide, *p* = 0.0038) (Figure 9C). GSH levels did not differ significantly between the experimental groups (Figure 9B).

### 3.6. Effects of GLP-1RAs on Oxidative Stress Parameters in Coronary Venous Effluent

**Figure 10:**
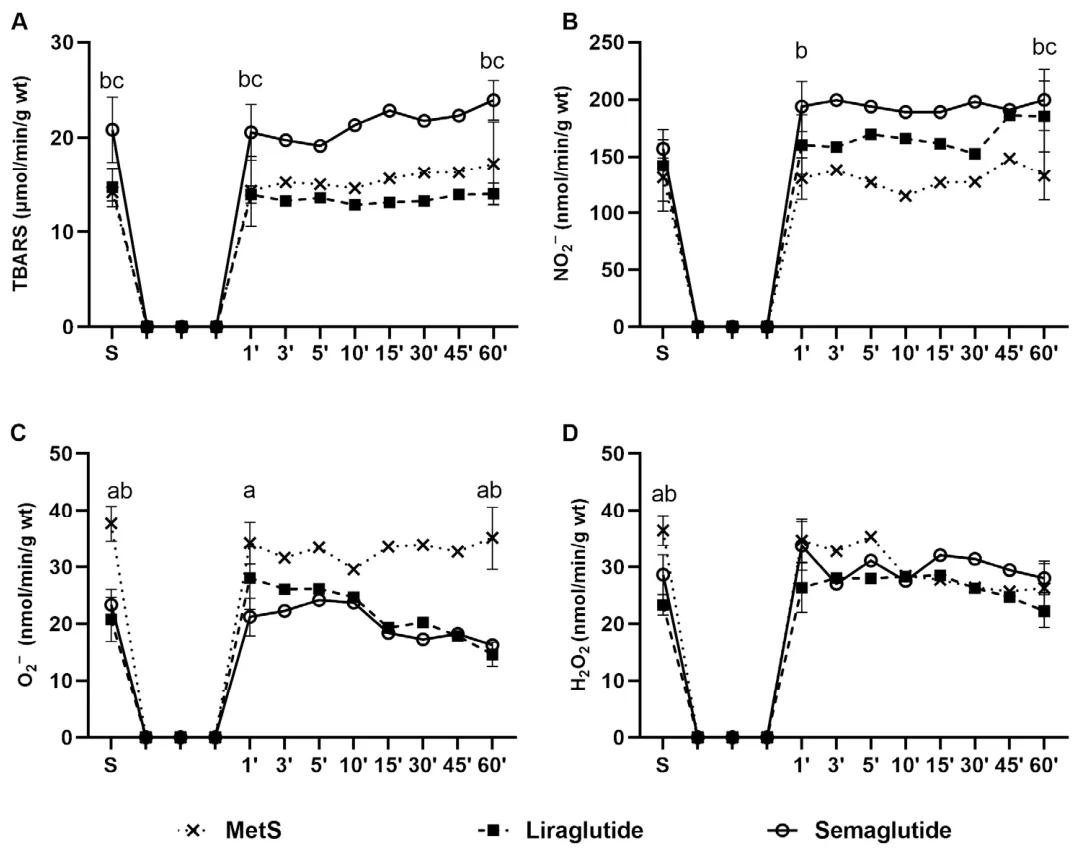
Effects of liraglutide and semaglutide on pro-oxidant levels in CVE between experimental groups. (**A**) TBARS; (**B**) NO_2_^−^; (**C**) O_2_^−^; (**D**) H_2_O_2_. Between-group comparisons at three time points. S—end of the stabilization period; 1′ and 60′—1st and 60th minute of reperfusion. a—MetS vs. liraglutide; b—MetS vs. semaglutide; c—liraglutide vs. semaglutide. Statistical significance at the level of *p* < 0.05. Two-way repeated-measures ANOVA with Šídák multiple-comparisons test. Values are presented as mean ± SD, n = 10 per group.

Pro-oxidative markers measured in the coronary venous effluent (CVE) are presented in Figure 10. GLP-1RAs produced distinct effects on oxidative stress markers.

For TBARS, semaglutide treatment resulted in a significant increase in this pro-oxidative marker compared with the MetS group at all investigated time points, including stabilization and both early and late reperfusion (stabilization, *p* = 0.0052; 1st minute, *p* = 0.0110; 60th minute, *p* = 0.0047), as well as compared with the liraglutide-treated group (*p* = 0.0132, *p* = 0.0055 and *p* < 0.0001, respectively), whereas liraglutide did not significantly affect TBARS (Figure 10A).

Semaglutide also increased NO_2_^−^ release compared with MetS during early and late reperfusion (*p* = 0.0046 and *p* = 0.0024, respectively) and compared with liraglutide at late reperfusion (*p* = 0.0114) (Figure 10B).

Liraglutide significantly reduced O_2_^−^ release compared with the MetS group at all time points (stabilization, early, and late reperfusion) (all *p* < 0.0001), while semaglutide decreased O_2_^−^ at stabilization and the 60th minute of reperfusion (both *p* < 0.0001) (Figure 10C).

Both treatments reduced H_2_O_2_ release during stabilization compared with MetS (liraglutide, *p* < 0.0001; semaglutide, *p* = 0.0188) (Figure 10D).

**Figure 11:**
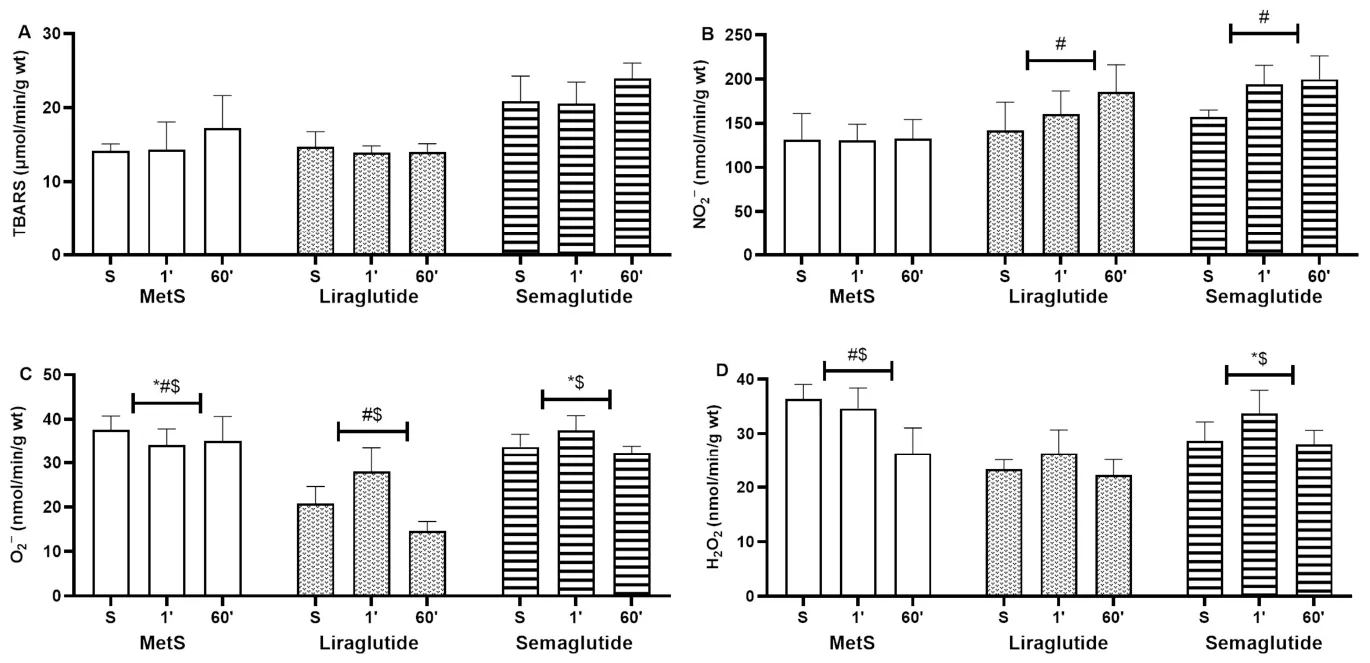
Effects of liraglutide and semaglutide on pro-oxidant levels between specific time points in the experimental group in CVE. (**A**) TBARS; (**B**) NO_2_^−^; (**C**) O_2_^−^; (**D**) H_2_O_2_. S—end of the stabilization period; 1′ and 60′—1st and 60th minutes of reperfusion. Within-group comparisons between time points: * S vs. 1′; # S vs. 60′; $ 1′ vs. 60′. Statistical significance at the level of *p* < 0.05. Two-way repeated-measures ANOVA with Šídák multiple-comparisons test. Values are presented as mean ± SD, n = 10 per group.

The values of pro-oxidative parameters measured in the CVE of isolated rat hearts in the control and experimental groups after the stabilization period and during the 1st and 60th minute of reperfusion are presented in Figure 11.

The TBARS remained unchanged in all groups and at all time points of interest (stabilization, 1st, and 60th minute of reperfusion) (Figure 11A).

On the other hand, administration of both liraglutide and semaglutide resulted in a significant increase in NO_2_^−^ levels at the 60th minute of reperfusion compared with the stabilization period (*p* = 0.0425, *p* = 0.0324) (Figure 11B).

Time-dependent changes were also observed for O_2_^−^. Following liraglutide treatment, O_2_^−^ release was highest at the 1st minute of reperfusion, exceeding both the stabilization value (*p* = 0.0425) and the value at the 60th minute of reperfusion (*p* < 0.0001). A similar effect was observed in the semaglutide group, with an increase in the 1st minute of reperfusion (*p* = 0.0343, *p* = 0.0020), while in the MetS group, the highest value was in the stabilization period (*p* = 0.0023, *p* = 0.0120) (Figure 11C).

Regarding H_2_O_2_ release, MetS animals without treatment exhibited a significant reduction in H_2_O_2_ concentration at the 60th minute of reperfusion compared with both the 1st minute and the stabilization period (*p* = 0.0023, *p* = 0.0043). On the other hand, in the semaglutide group, higher levels were observed in the first minute of reperfusion compared to stabilization (*p* = 0.0050) and the last minute of reperfusion (*p* = 0.0251) (Figure 11D).

### 3.7. Effects of GLP-1RAs on Heart Morphology

**Figure 12:**
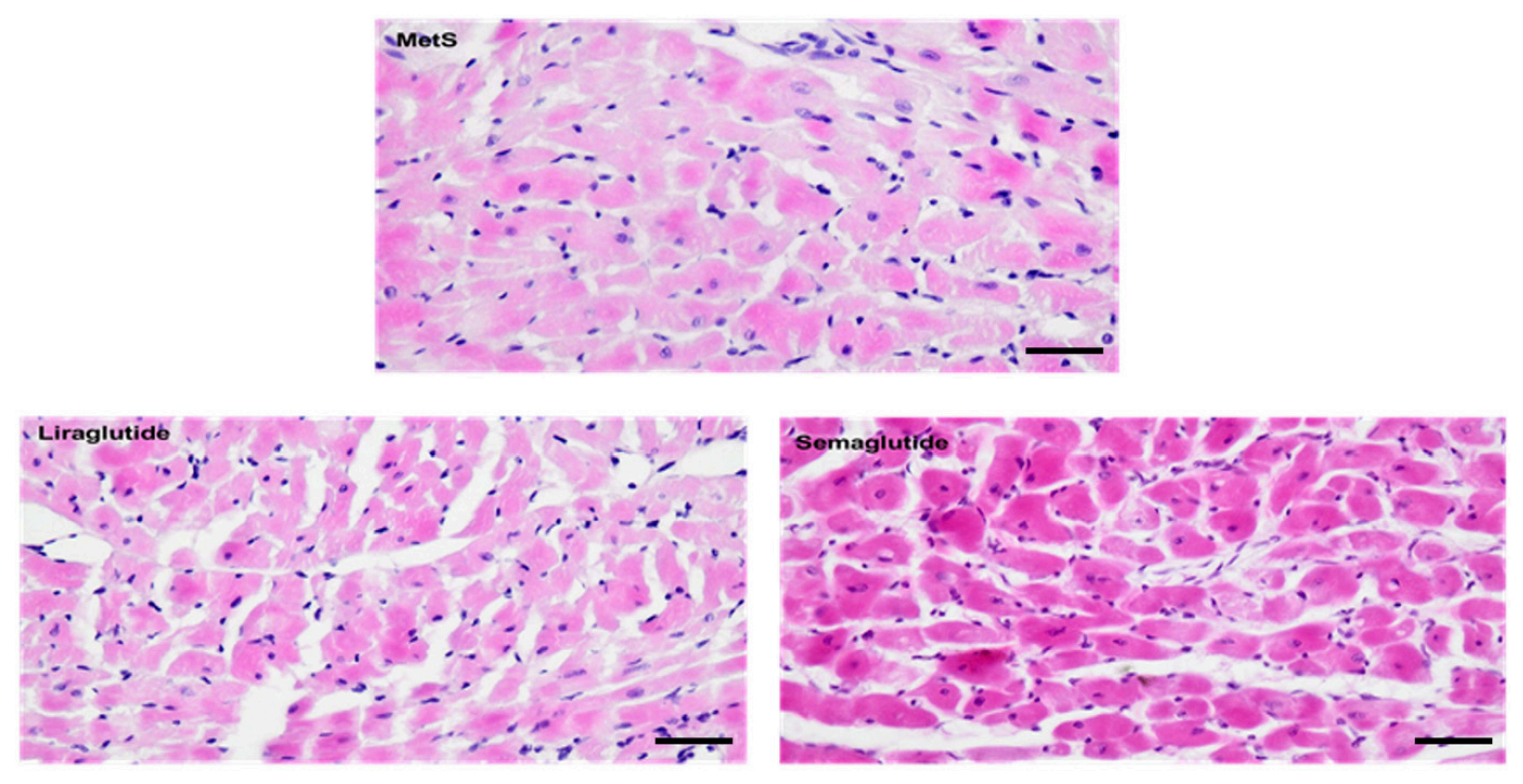
Representative micrograph of HE-stained cardiac tissue from rats with metabolic syndrome treated with liraglutide or semaglutide. Total magnification 40×, scale bar = 20 µm. All sections were stained in the same run and imaged under identical acquisition settings.

Histological sections of rat hearts are presented in Figure 12. In the MetS group, pronounced myocardial inflammation was observed, characterized by inflammatory cell infiltration, expanded interstitial space, wavy myocardial fibers, and cardiomyocyte hypertrophy. In the liraglutide-treated group, an expanded interstitial space, cardiomyocyte hypertrophy, and wavy myocardial fibers were observed, without evidence of inflammatory cell infiltration. In the semaglutide-treated group, histological analysis revealed an expanded interstitial space, wavy myocardial fibers, and cardiomyocyte hypertrophy accompanied by hypertrophy of individual nuclei, also in the absence of inflammatory infiltration.

**Figure 13:**
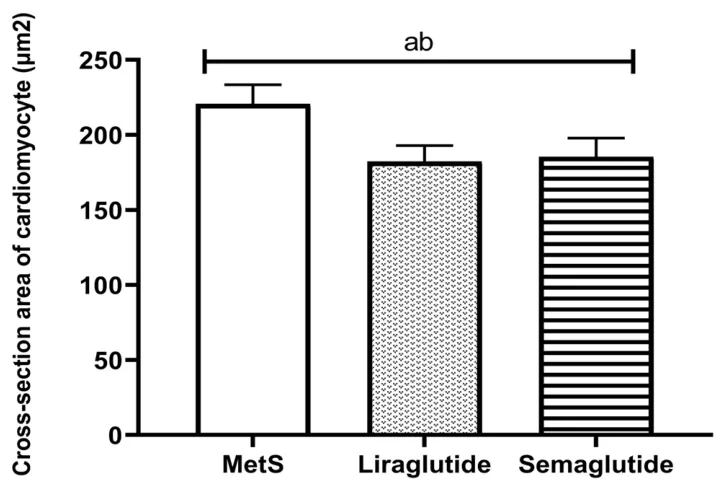
Effects of liraglutide and semaglutide on cross-sectional area of cardiomyocytes. a—MetS vs. liraglutide; b—MetS vs. semaglutide. Statistical significance at the level of *p* < 0.05. One-way ANOVA with Tukey’s multiple-comparisons test. Values are presented as mean ± SD, n = 10 per group.

Morphometric analysis was performed to assess changes in cardiomyocyte cross-sectional area following completion of the experimental protocol, and the results are presented in Figure 13. Treatment with GLP-1 receptor agonists led to a reduction in cardiomyocyte cross-sectional area compared with the MetS group.

**Figure 14:**
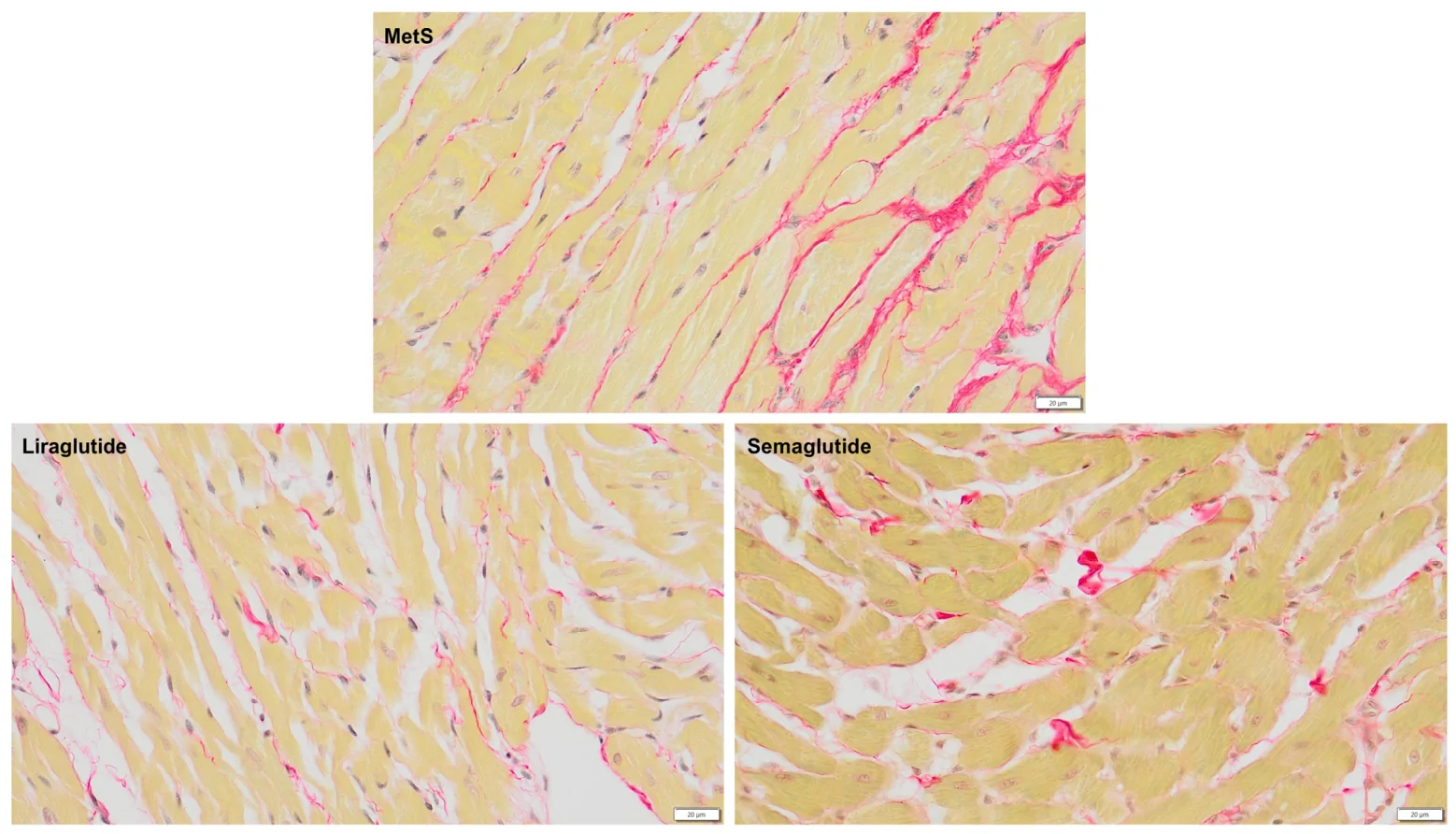
Representative micrographs of Picrosirius Red-stained cardiac tissue sections from rats with metabolic syndrome treated with liraglutide or semaglutide. Objective magnification 40×, scale bar = 20 µm. All sections were stained in the same run and imaged under identical acquisition settings.

**Figure 15:**
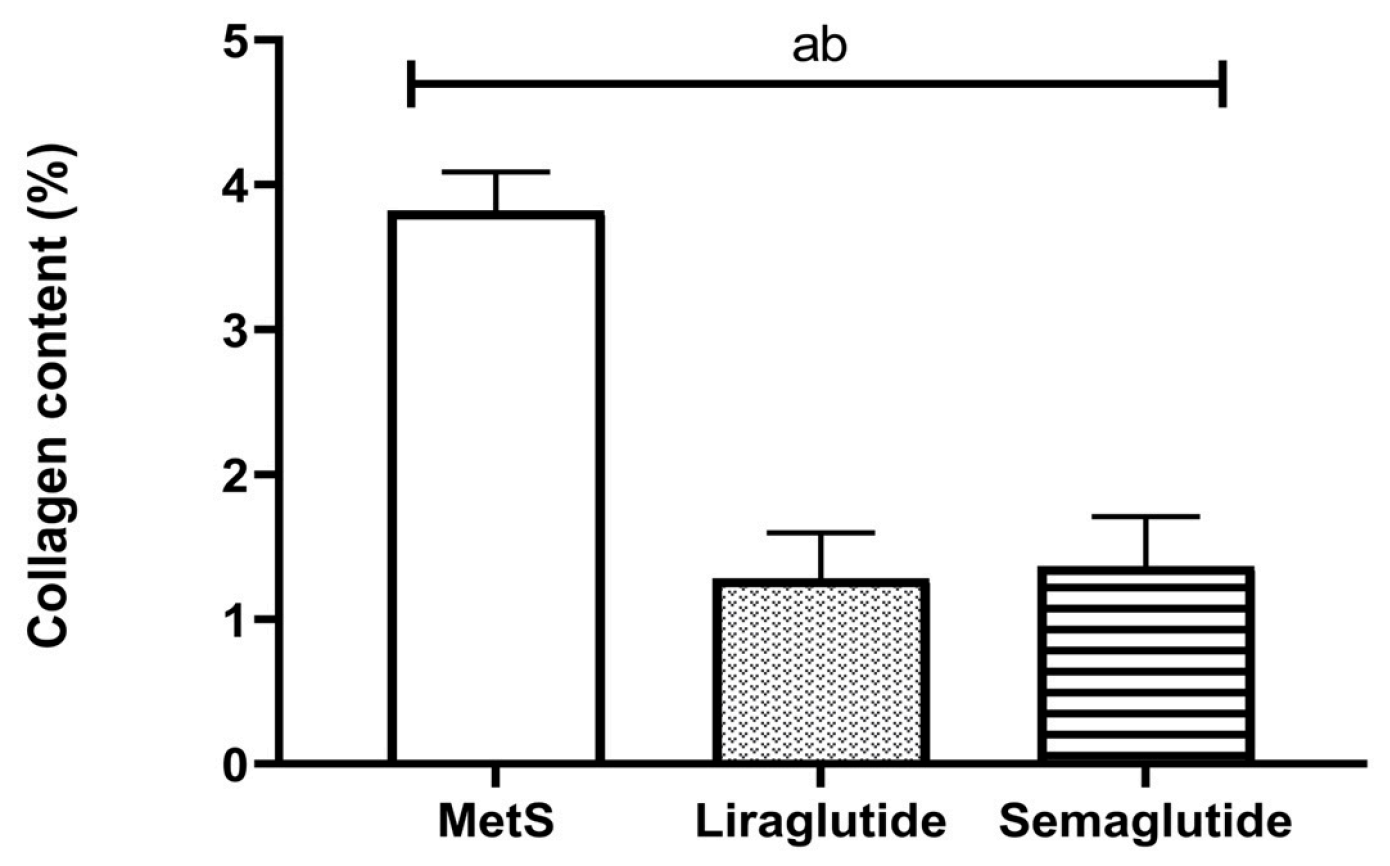
Effects of liraglutide and semaglutide on myocardial collagen content. a—MetS vs. liraglutide; b—MetS vs. semaglutide; Statistical significance at the level of *p* < 0.05. One-way ANOVA with Tukey’s multiple-comparisons test. Values are presented as mean ± SD, n = 10 per group.

The changes in myocardial collagen content following completion of the experimental protocol are presented in Figure 14 and Figure 15. A reduction in collagen fiber content was observed in both experimental groups compared with the MetS group. Collagen fiber deposits in rat heart tissue were visualized using Picrosirius Red staining, appearing as red-stained areas. In the MetS group, pronounced collagen deposition was observed, characterized by thicker and more densely interconnected fibers. In contrast, treatment with both liraglutide and semaglutide significantly reduced myocardial collagen deposition, as clearly demonstrated in Figure 15.

### 3.8. Effects of GLP-1RAs on Liver Morphology

**Figure 16:**
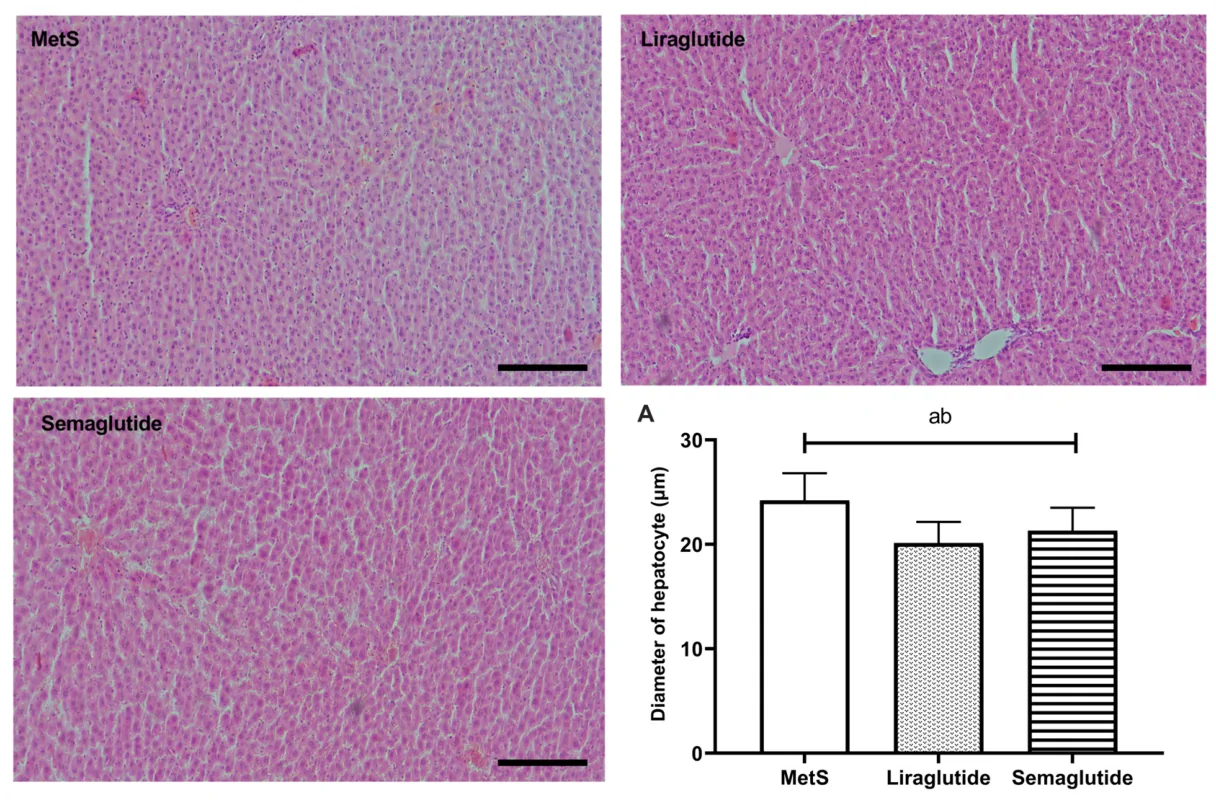
Histological and morphometric assessment of liver tissue in rats with metabolic syndrome treated with liraglutide or semaglutide. Representative hematoxylin and eosin (H&E)-stained liver sections (total 20× magnification, scale bar = 50 µm) from metabolic syndrome (MetS), liraglutide, and semaglutide groups. The accompanying graph (**A**) shows morphometric analysis of hepatocyte diameter, expressed as a percentage relative to the MetS group (MetS = 100%). a—MetS vs. liraglutide; b—MetS vs. semaglutide. Statistical significance at the level of *p* < 0.05. One-way ANOVA with Tukey’s multiple-comparisons test. Values are presented as mean ± SD, n = 10 per group.

Histological sections of liver tissue are presented in Figure 16. In the MetS group, discrete inflammation was observed, accompanied by vacuolization of individual hepatocytes and focal hemorrhages. In the experimental groups treated with liraglutide and semaglutide, similar histopathological alterations were detected, but inflammatory infiltration was not present in either treatment group. Morphometric analysis of hepatocyte diameter showed that treatment with liraglutide and semaglutide reduced hepatocyte diameter by 13% and 12%, respectively, compared with the MetS group. The difference in hepatocyte diameter between the semaglutide and liraglutide groups was not statistically significant.

### 3.9. Effects of GLP-1RAs on Pancreas Morphology

**Figure 17:**
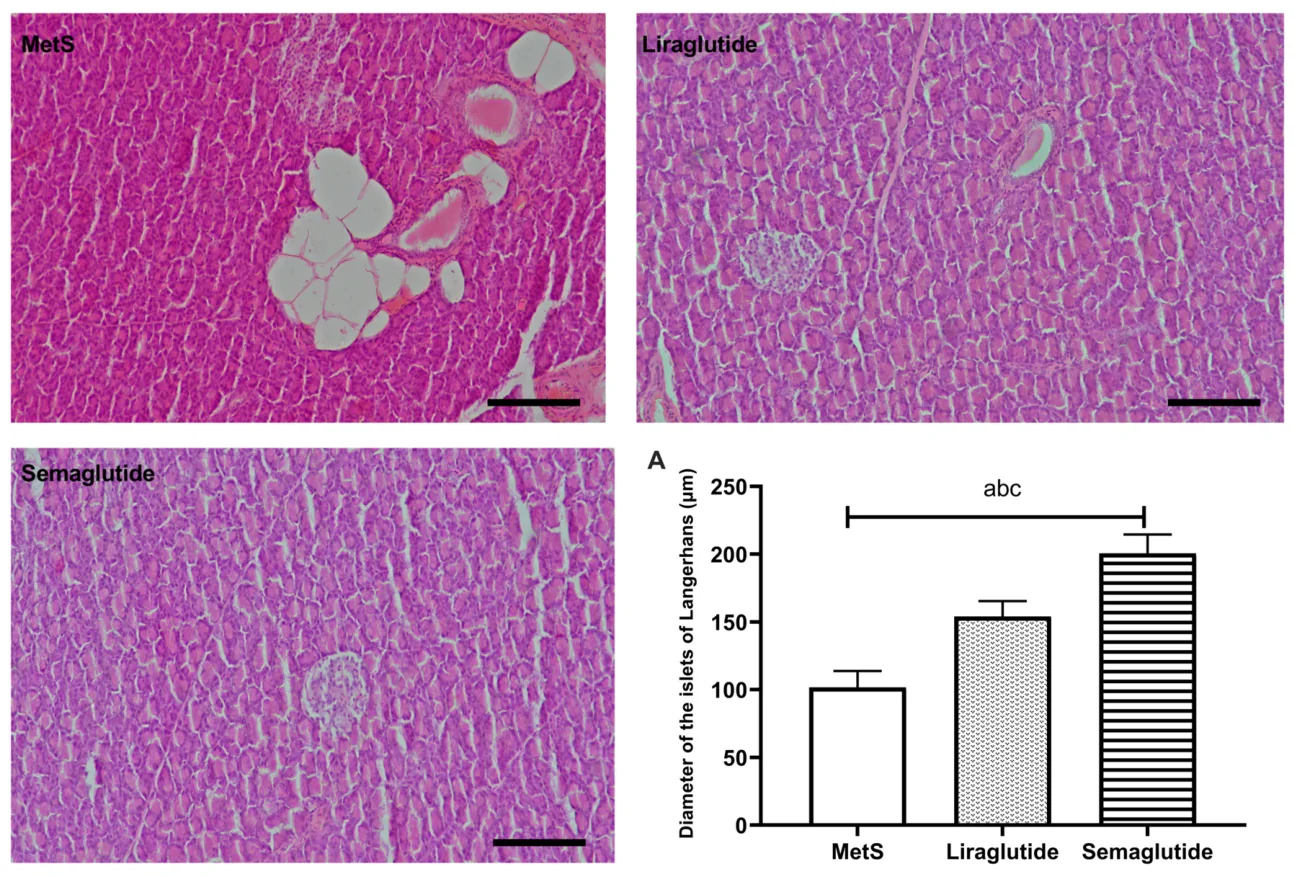
Histological and morphometric assessment of pancreatic tissue in rats with metabolic syndrome treated with liraglutide or semaglutide. Representative hematoxylin and eosin (H&E)-stained pancreatic sections (20× magnification; scale bar = 50 µm) from the metabolic syndrome (MetS), liraglutide, and semaglutide groups. The accompanying graph (**A**) shows morphometric analysis of the diameter of the islets of Langerhans, expressed as a percentage relative to the MetS group (MetS = 100%). a—MetS vs. liraglutide; b—MetS vs. semaglutide; c—liraglutide vs. semaglutide. Statistical significance at the level of *p* < 0.05. One-way ANOVA with Tukey’s multiple-comparisons test. Values are presented as mean ± SD, n = 10 per group.

Histological sections of pancreatic tissue are presented in Figure 17. In the MetS group, inflammatory changes were observed, accompanied by the presence of lipid droplets, while the pancreatic islets remained structurally preserved. In the liraglutide-treated group, preserved pancreatic morphology was observed, with no evidence of inflammation or lipid droplet accumulation. Similarly, in the semaglutide-treated group, intact pancreatic islet architecture was noted, without signs of inflammation or fatty deposits. The diameter of the islets of Langerhans increased by 51% in the liraglutide group and by 97% in the semaglutide group compared with the MetS group. Treatment with semaglutide increased the diameter of the islets of Langerhans by 30% compared with liraglutide (Figure 17).

## 4. Discussion

The aim of this study was to assess the cardioprotective potential of liraglutide and semaglutide in an experimental model of MetS in rats subjected to ischemia and reperfusion, with particular focus on changes in redox balance, in vivo and ex vivo cardiac function, and the histomorphology of the myocardium, liver, and pancreas. The metabolic alterations observed in this model were associated with cardiometabolic dysfunction, altered redox balance, and histopathological changes in the heart, liver, and pancreas. Treatment with liraglutide and semaglutide attenuated these metabolic, oxidative, functional, and structural alterations, indicating a protective effect of GLP-1 receptor agonism against the consequences of MetS. Notably, the two treatments showed distinct effects across the evaluated parameters, indicating that their cardiometabolic benefits may involve partially different mechanisms: semaglutide demonstrated a more pronounced effect on body weight regulation and systemic antioxidant response, whereas liraglutide exhibited a more favorable profile in certain aspects of coronary oxidative stress and post-ischemic myocardial recovery. The findings are considered below in three interconnected steps: the metabolic alterations of the model and their modification by treatment; their relationship with cardiac dysfunction and myocardial ischemia/reperfusion injury; and the contribution of oxidative stress and structural remodeling to these outcomes.

The effects of GLP-1RAs on myocardial I/R injury in the setting of MetS have become an important topic in contemporary cardiometabolic research. MetS, characterized by visceral adiposity, insulin resistance, impaired glucose tolerance, dyslipidemia, and hypertension, markedly increases cardiovascular risk and remains a major contributor to morbidity and mortality worldwide [26]. Although therapeutic options for cardiovascular disease and MetS have expanded, many still fail to fully prevent long-term complications, largely because the underlying pathophysiology remains incompletely understood. In this context, GLP-1RAs, particularly liraglutide and semaglutide, have emerged as clinically relevant agents because they improve glycemic control, support weight reduction, and may also exert direct cardiovascular and tissue-protective effects through broader metabolic, endothelial, and anti-inflammatory actions [12,27]. This broader protective profile is increasingly recognized across the GLP-1 receptor agonist class as a whole [15].

The present study contributes to this field by examining the effects of GLP-1 receptor agonist treatment on cardiac function and systemic homeostasis in an experimental model of MetS complicated by I/R injury. Its relevance lies not only in the assessment of in vivo and ex vivo cardiac performance, but also in the analysis of systemic and tissue oxidative stress, together with histomorphological changes in the heart, liver, and pancreas, thereby providing a broader view of the cardiometabolic and multiorgan consequences of treatment.

### 4.1. Metabolic Alterations in the Experimental Model of Metabolic Syndrome

Body weight is one of the defining features of MetS and was therefore monitored throughout the experimental protocol. In the untreated MetS group, body weight increased progressively during the six-week period, as expected for this model. However, semaglutide was the only agent associated with the slower weight gain during the study period. This finding is in line with clinical evidence showing substantial body weight reduction with semaglutide therapy, which has positioned it among the most potent GLP-1 receptor agonists in this respect [28]. Liraglutide has also been shown to reduce body weight, although usually to a lesser extent than semaglutide [29]. Mechanistically, these effects are linked to delayed gastric emptying, enhanced satiety, and reduced appetite mediated through vagal and central GLP-1 receptor pathways [13,30]. In our study, the absence of an overt reduction in body weight is most likely related to the young age of the animals, since physiological growth during the protocol may have masked treatment-associated effects on body mass.

Because MetS is strongly associated with insulin resistance, glycemia and glucose handling during the oral glucose tolerance test were also assessed. During chronic treatment, both liraglutide and semaglutide showed a downward trend in fasting glycemia, although intergroup differences did not reach statistical significance. During the oral glucose tolerance test, glucose values were consistently higher in the untreated MetS group than in the treated groups, indicating a less favorable glycemic response in the absence of therapy. These findings are in accordance with the well-established glucose-lowering action of GLP-1RAs and with previous studies demonstrating their beneficial role in glycemic control and glucose handling, particularly with longer-term treatment [28,29].

Dyslipidemia is another core component of MetS, and the lipid profile was therefore an important part of this study. Total cholesterol was significantly lower in both the liraglutide- and semaglutide-treated groups than in the untreated MetS group, indicating a beneficial effect on lipid metabolism and supporting earlier reports on favorable lipid-modifying actions of GLP-1RAs [31]. Triglyceride values were markedly elevated in the untreated MetS group, while treatment reduced triglycerides in both liraglutide- and semaglutide-treated animals. Rather than clearly separating the two agents, our results suggest that both exerted a favorable overall effect on lipid homeostasis, with semaglutide showing a particularly favorable pattern in the present model. Recent evidence also suggests that semaglutide may improve cardiometabolic risk beyond the degree expected from weight loss alone, supporting the possibility of direct metabolic and vascular effects [32]. Several mechanisms may account for these findings. Reduced appetite and caloric intake may indirectly improve the lipid profile, whereas direct hepatic actions of GLP-1 signaling may reduce very low-density lipoprotein (VLDL) production, suppress triglyceride synthesis, limit de novo lipogenesis, and promote fatty acid oxidation [33]. In addition, liraglutide has been shown to enhance reverse cholesterol transport, thereby improving hepatic and peripheral cholesterol handling [34].

Rats with MetS exhibited persistently elevated systolic blood pressure throughout the experimental period, which is consistent with previous reports obtained in different experimental models of MetS [16]. Treatment with liraglutide and semaglutide produced significant reductions in systolic blood pressure, with effects becoming evident already after several weeks of chronic administration. These findings agree with clinical and meta-analytic data showing that GLP-1RAs generally induce modest but potentially meaningful reductions in systolic blood pressure, particularly in high-risk cardiometabolic populations [35]. Although weight loss undoubtedly contributes to this effect, the relatively early onset observed in our study suggests the involvement of additional weight-independent mechanisms [36]. Experimental data support several complementary pathways, including improved endothelial nitric oxide synthase activity, vasorelaxation, natriuresis, reduced vascular stiffness, and attenuation of sympathetic activation [37,38]. Recent reviews have emphasized that these combined actions make GLP-1RAs particularly relevant in obesity-related hypertension and metabolic syndrome [39]. Because glycemic control, lipid metabolism, and blood pressure jointly determine myocardial oxygen demand, coronary perfusion, and afterload, the metabolic improvements described above provide a plausible basis for the cardiac and coronary findings addressed in the following section.

### 4.2. Relationship Between the Metabolic Phenotype, Cardiac Dysfunction, and Ischemia/Reperfusion Injury

Echocardiographic assessment suggested that treatment attenuated some of the adverse structural changes associated with MetS. Both liraglutide and semaglutide were associated with reduced interventricular septal and posterior wall hypertrophy, indicating partial protection against cardiac remodeling. At the same time, changes in ejection fraction were less pronounced and should be interpreted more cautiously. This is in keeping with the wider literature, where clinical and experimental findings have not always been entirely concordant. For example, clinical studies in heart failure have shown that liraglutide does not necessarily improve left ventricular ejection fraction despite possible structural or metabolic benefits. On the other hand, experimental studies have demonstrated reduced ventricular dimensions and improved function in some settings of myocardial hypertrophy [40]. These differences likely reflect differences in disease stage, treatment duration, and model-specific pathophysiology. Overall, our echocardiographic findings suggest that liraglutide and semaglutide exert a favorable influence on cardiac remodeling, even when systolic indices are not uniformly affected.

The ex vivo I/R experiments provided more direct evidence of cardioprotection. Hearts from untreated MetS animals displayed markedly impaired left ventricular contractility and relaxation during reperfusion, whereas liraglutide- and semaglutide-treated hearts showed better recovery of dp/dt max, dp/dt min, and systolic left ventricular pressure. By the end of reperfusion, these parameters were closer to baseline values in treated hearts than in untreated MetS animals, indicating improved post-ischemic functional recovery. These findings are consistent with the growing literature showing that GLP-1RAs can limit myocardial dysfunction during I/R by preserving mitochondrial integrity, optimizing calcium handling, and attenuating apoptosis and electrical instability [41]. Mechanistic studies by other investigators have implicated semaglutide in antiapoptotic signaling in experimental myocardial injury, with effects involving PKG/PKCε/ERK1/2-related pathways [42]. In parallel, previous work in other cardiovascular models has suggested that liraglutide may activate the eNOS/sGC/cGMP/PKG pathway [43]. Similar cardioprotective patterns have also been described for other GLP-1RAs, supporting the interpretation that myocardial protection may represent a class-related property, although the intensity and timing of the effect appear to vary between individual agents [44,45]. Because the isolated heart in the Langendorff experiments was perfused with a standard crystalloid solution and was no longer exposed to the systemic metabolic environment, this functional recovery points to a change acquired within the myocardium itself during the six weeks of treatment. The following section considers the redox and structural correlates of this change.

### 4.3. Contribution of Oxidative Stress and Histopathological Alterations

Oxidative stress represents one of the major mechanisms driving tissue injury in MetS and exacerbating ischemia/reperfusion damage. In the present study, systemic TBARS and NO_2_^−^ values did not change significantly, whereas O_2_^−^ was lower in both treated groups, with the greater reduction in the semaglutide group. Since O_2_^−^ is one of the main reactive oxygen species involved in mitochondrial dysfunction, endothelial damage, and apoptosis, this reduction is biologically meaningful [46]. The observed decrease in O_2_^−^, together with the increase in SOD activity, suggests that GLP-1 receptor agonists may improve antioxidant defense rather than simply suppress all oxidative markers uniformly [47]. Semaglutide showed the most pronounced effect on SOD, which is consistent with previous studies suggesting activation of the nuclear factor erythroid 2-related factor 2 (Nrf2) and sirtuin 1 (SIRT1) pathways. Both pathways are closely related to antioxidant gene transcription, mitochondrial protection, and redox homeostasis [48]. Experimental evidence also indicates that semaglutide can reduce oxidative injury and improve antioxidant enzyme activity in other tissues, supporting a broader cytoprotective role [49]. CAT activity was also increased in treated groups, indicating an adaptive strengthening of antioxidant defenses. However, this was not accompanied by a corresponding reduction in H_2_O_2_ levels, suggesting that hydrogen peroxide handling in this model may depend on additional factors beyond catalase alone [50].

However, systemic measurements may not fully reflect the dynamic redox changes occurring within the myocardium and coronary circulation during ischemia/reperfusion. To further characterize these local effects, analysis of the coronary venous effluent provided additional insight into changes in the redox environment during reperfusion. Both liraglutide and semaglutide were associated with increased NO_2_^−^ values during reperfusion, which may be consistent with altered nitric oxide metabolism or increased nitric oxide bioavailability at the coronary level. Given the role of nitric oxide in endothelial function and myocardial perfusion, these findings may suggest a potential improvement in coronary endothelial responsiveness, although this interpretation should be considered exploratory. O_2_^−^ patterns differed somewhat between the two agents. Liraglutide showed a transient early reperfusion increase followed by a decline below baseline levels, whereas semaglutide produced a smaller reduction in O_2_^−^ than liraglutide. H_2_O_2_ behavior suggested active superoxide dismutation, particularly in the liraglutide group. These findings are compatible with previous data showing that liraglutide can inhibit NADPH oxidase activity and increase antioxidant enzyme expression in endothelial and cardiac cells [51]. Semaglutide has been linked to increased total SOD activity, AMPK activation, and suppression of inflammatory signaling in myocardial stress [18].

Histomorphological analysis provided strong evidence of multiorgan protection. In the heart, untreated MetS animals exhibited cardiomyocyte hypertrophy, inflammatory infiltrates, interstitial expansion, and increased collagen deposition, all of which are characteristic of maladaptive remodeling under chronic metabolic stress. Both liraglutide and semaglutide substantially reduced myocardial collagen content and attenuated cardiomyocyte hypertrophy, indicating anti-remodeling and antifibrotic effects. These findings are in line with previous data suggesting that GLP-1RAs reduce myocardial fibrosis through modulation of TGF-β/Smad3 signaling and extracellular matrix turnover [52]. They are also broadly consistent with cardiovascular outcome data from the LEADER and SUSTAIN programs, which support the cardiovascular benefit of liraglutide and semaglutide in high-risk populations [53,54].

Extracardiac tissues showed a similar pattern. In the liver, both liraglutide and semaglutide improved histological appearance by reducing inflammatory changes, although mild vacuolization persisted, suggesting partial rather than complete reversal of hepatic metabolic injury [55,56]. Consistent with this, hepatocyte diameter was about 12–13% smaller in both treated groups. In MetS, hepatocyte enlargement mainly reflects intracellular lipid accumulation, and swollen, fat-laden hepatocytes compress the sinusoids [57]. The smaller hepatocytes therefore point to a lower hepatocellular lipid load, in agreement with the attenuation of hepatocellular hypertrophy by liraglutide in rats with metabolic syndrome [16] and the reduction in lipid-laden and ballooned hepatocytes by semaglutide in obese mice [58]. Because hepatocytes lack functionally relevant GLP-1 receptor expression, this effect is most likely indirect, mediated by reduced caloric intake and adiposity and improved insulin sensitivity [12,53]. In the pancreas, both agents were associated with reduced inflammatory changes and diminished lipid accumulation, which is likely related to their beneficial effects on body weight regulation, glucose handling, and lipid metabolism [30]. Moreover, the diameter of the islets of Langerhans was about 50% larger with liraglutide and almost twofold larger with semaglutide than in untreated MetS rats. In this model, islet size reflects the balance between streptozotocin-induced β-cell loss and compensatory expansion driven by insulin resistance. GLP-1RAs shift this balance toward β-cell survival by stimulating β-cell proliferation and suppressing apoptosis, an effect that is most pronounced in hyperglycemic animals with a β-cell deficit [59]. Accordingly, liraglutide restored islet size in diabetic db/db mice and semaglutide recovered islet size and β-cell mass in diet-induced obese mice [25,60]. Given that β-cells are particularly vulnerable to oxidative stress, the lower plasma superoxide levels and higher SOD activity observed in our treated animals may have contributed to the preservation of islet structure. However, islet diameter on histological sections is not a direct measure of β-cell mass, and the effect of these agents on β-cell mass depends strongly on the metabolic context—in normoglycemic rodents they may even reduce it—so this finding should be interpreted with caution [61].

### 4.4. Limitations of the Study

This study has several limitations. First, given that we assessed the cardioprotective effects of semaglutide and liraglutide in rats with MetS, a group of healthy animals was not included. Comparison with healthy, non-MetS animals might provide a more complete and clearer interpretation of the results obtained. Second, we applied only one dose of both drugs, while assessment of the effects of two or more doses could identify the dose (or doses) with the greatest cardioprotective potential, with the potential for further translational research. Third, after randomization of rats to experimental groups, no additional blinding measures of the researchers during drug administration and analysis of the results were used. To minimize bias, outcome assessment relied on objective measurements and standardized procedures.

## 5. Conclusions

In an experimental model of metabolic syndrome complicated by myocardial ischemia/reperfusion injury, six weeks of treatment with liraglutide or semaglutide improved the metabolic profile, attenuated myocardial hypertrophy and fibrosis, reduced inflammatory and lipid changes in the liver and pancreas, and improved post-ischemic recovery of contractility and relaxation. The two agents differed in profile: semaglutide was more effective in limiting weight gain and enhancing systemic antioxidant capacity, whereas liraglutide had the more pronounced effect on coronary redox regulation and functional recovery during late reperfusion. These findings indicate that in metabolic syndrome, the benefit of GLP-1 receptor agonism is not confined to metabolic control but extends to the myocardial response to ischemia/reperfusion, and that the two agents may not be interchangeable in this respect. The overall consistency of these findings with preclinical and emerging clinical evidence strengthens the rationale for further translational investigation of liraglutide and semaglutide as potential therapeutic strategies for reducing cardiovascular and broader metabolic complications associated with metabolic syndrome.

## Figures and Tables

**Table 1:** Effects of liraglutide and semaglutide on echocardiographic parameters.

	MetS	Liraglutide	Semaglutide
IVSd (cm)	0.19 ± 0.02	0.12 ± 0.01 **^a^**	0.14 ± 0.01 **^b^**
LVIDd (cm)	0.69 ± 0.03	0.73 ± 0.03	0.78 ± 0.03 **^b,c^**
LVPWd (cm)	0,17 ± 0.01	0.15 ± 0.04	0.16 ± 0.01
IVSs (cm)	0.21 ± 0.03	0.19 ± 0.01	0.20 ± 0.01
LVIDs (cm)	0.41 ± 0.03	0.43 ± 0.03 **^a^**	0.44 ± 0.08 **^b,c^**
LVPWs (cm)	0.21 ± 0.02	0.20 ± 0.03	0.19 ± 0.02
FS (%)	41.07 ± 3.76	41.87 ± 2.36	42.35 ± 3.39
EF (%)	78.58 ± 4.08	76.98 ± 2.69	79.71 ± 8.41

Interventricular septal wall thickness at end-systole (IVSs) and end-diastole (IVSd); left ventricular internal diameter at end-systole (LVIDs) and end-diastole (LVIDd); left ventricular posterior wall thickness at end-systole (LVPWs) and end-diastole (LVPWd); fractional shortening (FS); ejection fraction (EF); MetS: metabolic syndrome group; statistical significance at the level of *p* < 0.05. ^a^—MetS vs. liraglutide; ^b^—MetS vs. semaglutide; ^c^—liraglutide vs. semaglutide. Groups were compared by one-way ANOVA with a Tukey multiple-comparisons test. Values are presented as mean ± SD, n = 10 per group.

## Data Availability

The data presented in this study are available on request from the corresponding author, due to study-specific information that is subject to institutional considerations regarding data sharing.

## Abbreviations

The following abbreviations are used in this manuscript:

AMPK	AMP-activated protein kinase
ATP	adenosine triphosphate
CAT	catalase
CF	coronary flow
cGMP	cyclic guanosine monophosphate
CVE	coronary venous effluent
DLVP	diastolic left ventricular pressure
dp/dt max	maximum rate of left ventricular pressure development
dp/dt min	minimum rate of left ventricular pressure development
DTNB	5,5′-dithiobis-2-nitrobenzoic acid
EDTA	ethylenediaminetetraacetic acid
EF	ejection fraction
ELISA	enzyme-linked immunosorbent assay
eNOS	endothelial nitric oxide synthase
ERK1/2	extracellular signal-regulated kinase 1/2
FS	fractional shortening
GLP-1	glucagon-like peptide-1
GLP-1RA	glucagon-like peptide-1 receptor agonist(s)
GSH	reduced glutathione
H_2_O_2_	hydrogen peroxide
HE	hematoxylin and eosin
HR	heart rate
I/R	ischemia/reperfusion
IVSd	interventricular septal thickness at end-diastole
IVSs	interventricular septal thickness at end-systole
LV	left ventricular
LVIDd	left ventricular internal diameter at end-diastole
LVIDs	left ventricular internal diameter at end-systole
LVPWd	left ventricular posterior wall thickness at end-diastole
LVPWs	left ventricular posterior wall thickness at end-systole
MetS	metabolic syndrome
NADPH	nicotinamide adenine dinucleotide phosphate
NBT	nitro blue tetrazolium
NO_2_^−^	nitrite
Nrf2	nuclear factor erythroid 2-related factor 2
O_2_^−^	superoxide anion radical
OGTT	oral glucose tolerance test
PKCε	protein kinase C epsilon
PKG	protein kinase G
ROS	reactive oxygen species
SD	standard deviation
sGC	soluble guanylate cyclase
SIRT1	sirtuin 1
SLVP	systolic left ventricular pressure
Smad3	mothers against decapentaplegic homolog 3
SOD	superoxide dismutase
TBA	thiobarbituric acid
TBARS	thiobarbituric acid reactive substances
TGF-β	transforming growth factor-β
Tris-HCl	tris(hydroxymethyl)aminomethane hydrochloride
VLDL	very low-density lipoprotein
